# Metagenomic analysis reveals severity-dependent microbial succession and correlation with host inflammatory response in oral and maxillofacial space infections

**DOI:** 10.3389/fcimb.2025.1695928

**Published:** 2026-01-08

**Authors:** Xijun Wang, Lei Ye, Yimin Liu, Hui Li, Huan Shi, Lingyan Zheng

**Affiliations:** 1 Department of Oral Surgery, Shanghai Ninth People's Hospital, Shanghai Jiao Tong University School of Medicine, Shanghai, China; 2 College of Stomatology, Shanghai Jiao Tong University, Shanghai, China; 3 National Center for Stomatology, Shanghai, China; 4 National Clinical Research Center for Oral Diseases, Shanghai, China; 5 Shanghai Key Laboratory of Stomatology, Shanghai, China

**Keywords:** biomarker, inflammation, metagenomic next-generation sequencing, microbiome, oral and maxillofacial space infection

## Abstract

**Background:**

Oral and maxillofacial space infections (OMSI) vary widely in clinical severity, yet the relationships between microbial community patterns in the abscess niche and host inflammatory responses remain incompletely characterized.

**Methods:**

We conducted a retrospective, cross-sectional, severity-stratified study of 197 patients diagnosed with OMSI between January 2020 and November 2023. Patients were stratified into mild (n=90), moderate (n=41), and severe (n=66) groups based on established clinical criteria. We performed mNGS on abscess pus samples to characterize the microbial community composition and assessed associations between these features and systemic inflammatory markers.

**Results:**

Although α-diversity did not differ significantly among severity groups, β-diversity analysis revealed distinct microbial communities. Pairwise analyses indicated a threshold-like community shift, characterized by a significant divergence between mild and severe infections, while the moderate group exhibited an intermediate composition that overlapped with both. Severe infections were characterized by an enrichment of *Prevotella.* Furthermore, analysis of predominant taxa (>30% abundance) revealed considerable microbial heterogeneity, challenging a simple monoinfection model. Notably, a machine learning-identified microbial profile comprising *Streptococcus*, *Corynebacterium*, and *Pseudomonas* was significantly correlated with elevated systemic inflammatory markers.

**Conclusion:**

This study characterizes associations between abscess-site microbial communities and host inflammatory profiles across OMSI severity strata. Given the cross-sectional design and the lack of an external validation cohort, the present findings should be interpreted as exploratory and non-causal. Future multicenter prospective studies including independent validation cohorts are warranted to test reproducibility and to evaluate whether any candidate features possess generalizable predictive value.

## 1 Introduction

Oral and maxillofacial space infections (OMSI) are critical emergencies in oral and maxillofacial surgery, characterized by rapid progression and the potential to culminate in life-threatening complications such as descending necrotizing mediastinitis, intracranial infection, and sepsis if not promptly managed. The majority of OMSI cases are odontogenic, with other common etiologies including salivary gland infections and traumatic injuries (Zhou et al., 2023; Wang et al., 2025; Qian et al., 2021). Due to the complex anatomy of the fascial spaces, infections can spread swiftly, which necessitates early and accurate microbiological diagnosis to guide effective therapeutic interventions (Robertson et al., 2015; Kitamura, 2018; Bigus et al., 2023). Although conventional culture-based methods remain the diagnostic gold standard, they are hampered by several limitations, including low sensitivity, prolonged turnaround times, and inadequate detection of anaerobic and fastidious organisms (Grumaz et al., 2019; Shi et al., 2023; Simner et al., 2018). Consequently, there is an urgent clinical need for more sensitive and comprehensive diagnostic approaches, such as metagenomic next-generation sequencing (mNGS), to enhance the management of OMSI.

To address this diagnostic gap, metagenomic next-generation sequencing (mNGS) has emerged as a powerful, culture-independent technology. By enabling unbiased, comprehensive profiling of microbial communities—including bacteria, fungi, and viruses—directly from clinical specimens within a clinically relevant timeframe, mNGS circumvents the inherent biases of culture-based techniques (Li, 2022; Angel et al., 2025; Han et al., 2019; Ding et al., 2024; Zhao et al., 2024; Greninger et al., 2017). Its application in OMSI has already shown promise, revealing a greater diversity of pathogens and demonstrating superior detection rates for anaerobic bacteria compared to traditional methods (Shi et al., 2023; Lei et al., 2025; Chen et al., 2024; Zhou et al., 2024).

While previous studies, including work by our group (Shi et al., 2023) which established the superior diagnostic yield of mNGS for pathogen identification in OMSI, have demonstrated the utility of mNGS in identifying a broader spectrum of OMSI-associated pathogens (Bigus et al., 2023; Li et al., 2022; Sultana et al., 2017), systematic investigations of the pus microbiome across the full spectrum of disease severity, from mild to severe, are notably absent. Furthermore, existing research has predominantly focused on qualitative pathogen identification, leaving the quantitative relationships between microbial community structure and host inflammatory responses largely unexplored. We therefore aimed to address this knowledge gap by employing mNGS to perform a comprehensive characterization of the pus microbiome in OMSI patients stratified by clinical severity. Our primary objective was to delineate distinct microbial profiles associated with disease severity and to correlate these findings with host inflammatory markers. To address this knowledge gap, we hypothesized that this integrative approach would not only elucidate critical aspects of OMSI pathophysiology but also establish a foundation for developing novel diagnostic tools and precision therapeutic strategies, ultimately improving patient outcomes.

## 2 Materials and methods

### 2.1 Study design and ethical approval

In this study included patients diagnosed with oral and maxillofacial space infections (OMSI) at the Department of Oral and Maxillofacial Surgery, Shanghai Jiao Tong University School of Medicine, Affiliated Ninth People’s Hospital, between January 2020 and November 2023. The study was conducted in accordance with the principles of the Declaration of Helsinki and received approval from the institutional Ethics Committee (Approval No. SH9H-2021-T400-2). Written informed consent was obtained from all participants prior to their inclusion.

**Figure 1:**
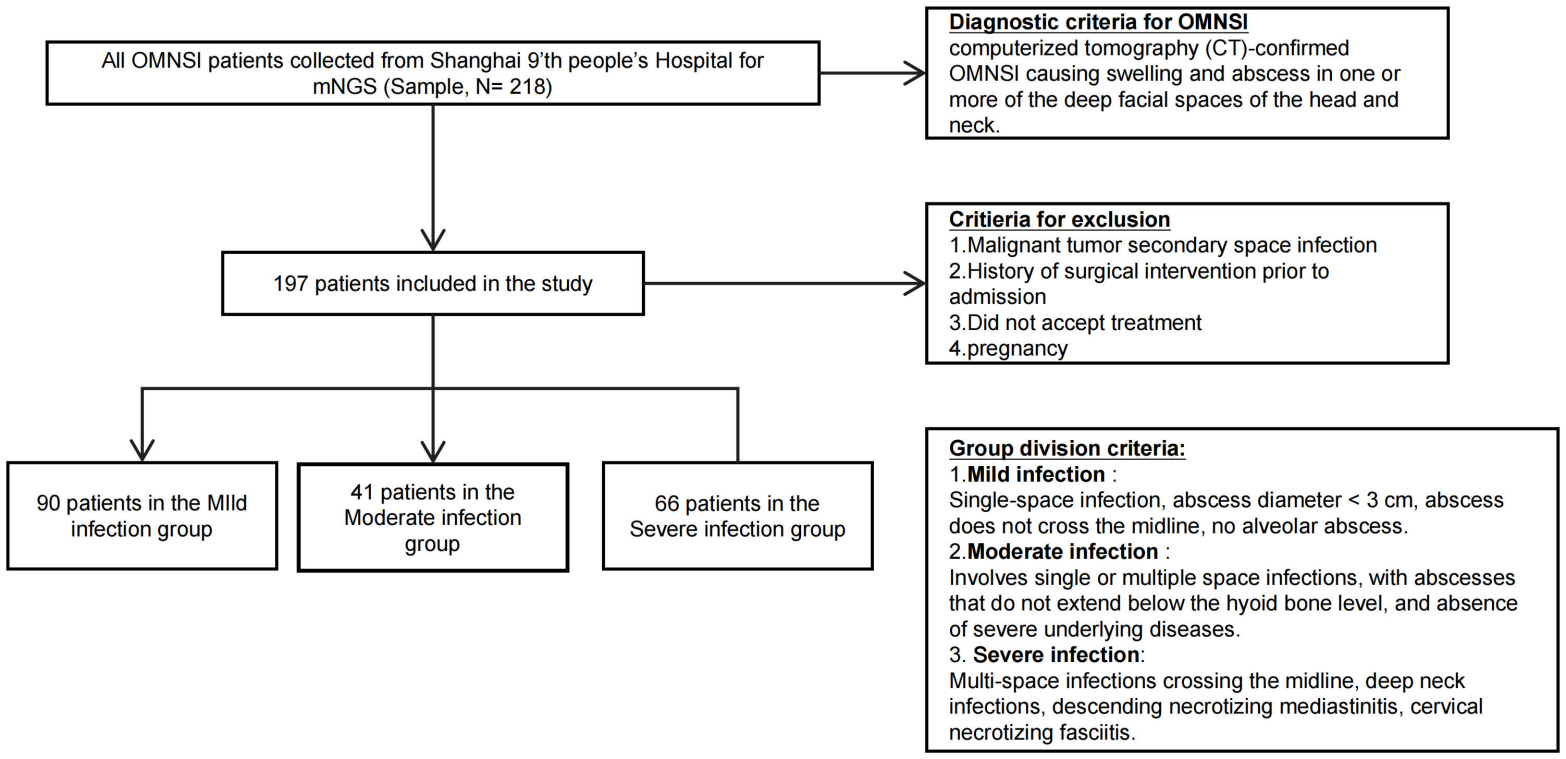
Inclusion and exclusion flowchart of study.

The diagnosis of OMSI was confirmed by computed tomography (CT) for all individuals. Patients were stratified into mild, moderate, or severe groups based on a previously established clinical classification system (Wang et al., 2024). Exclusion criteria included: (1) malignancy secondary to the infection; (2) a history of surgical intervention prior to admission; (3) refusal of treatment, or (4) pregnancy. These specific criteria were implemented to enhance the internal validity of the study by minimizing the influence of major confounding factors. The exclusion of patients with malignancy or prior surgical/antibiotic intervention was intended to create a more homogeneous cohort, thereby allowing a clearer assessment of the relationships between infection severity, the pus microbiome, and the host inflammatory response, independent of these potent confounding variables. Following the application of these criteria, a final cohort of 197 patients was selected for analysis, as detailed in the patient selection flowchart (**Figure 1**).

### 2.2 Clinical data collection and specimen workflow

A comprehensive set of clinical data for all 197 patients was retrospectively abstracted from Hospital Information System (HIS). This dataset included demographics (age and sex), comorbidities (e.g., diabetes, cardiovascular disease, hypertension), and clinical signs at admission (e.g., dyspnea, dysphagia, limited mouth opening, fever, and heart rate).

For microbiological analysis, abscess pus samples were collected from each patient by two experienced clinicians under strict aseptic conditions. Samples were immediately processed for metagenomic next-generation sequencing (mNGS) analysis.

In parallel with pus sample collection, peripheral blood was drawn from each patient within two hours of admission to comprehensively characterize the host's systemic response to infection. The analysis comprised three key components. First, hematological parameters were determined using a complete blood count (CBC) with differential, providing data on total white blood cells (WBC), and both the percentage and absolute counts of neutrophils (NEUT%, NEUT<ns/>) and lymphocytes (Lymph%, Lymph<ns/>). Second, established biomarkers of bacterial infection and inflammation, C-reactive protein (CRP) and procalcitonin (PCT), were quantified. Third, to gain deeper insight into the immunoregulatory and pro-inflammatory pathways, a panel of critical serum cytokines was measured, which included Interleukin-1 beta (IL-1β), Interleukin-2 receptor (IL-2R), Interleukin-6 (IL-6), Interleukin-8 (IL-8), and Tumor Necrosis Factor-alpha (TNF-α).

### 2.3 DNA extraction, library preparation, and sequencing

Genomic DNA (gDNA) was extracted from pus specimens using the QIAamp® UCP Pathogen DNA Kit (Qiagen, Hilden, Germany) according to the manufacturer’s protocol. Subsequently, sequencing libraries were prepared from the extracted gDNA with the Nextera XT DNA Library Prep Kit (Illumina, San Diego, CA, USA).

A two-step quality control (QC) process was performed. First, library concentration was quantified using the Qubit dsDNA HS Assay Kit (Thermo Fisher Scientific, Waltham, MA, USA). Second, library size distribution and integrity were assessed using an Agilent 2100 Bioanalyzer with a High Sensitivity DNA Kit (Agilent, Santa Clara, CA, USA). Libraries that passed QC were pooled and sequenced on an Illumina NextSeq 550Dx platform. Sequencing was performed in single-end mode for 75 cycles, generating a minimum of 20 million reads per library.

### 2.4 Statistical analysis

Statistical analyses were performed using R (version 4.2.3), IBM SPSS Statistics (version 26.0), and Python (version 3.9). Clinical and demographic characteristics were compared across severity groups using one-way ANOVA for normally distributed continuous variables (e.g., Age, BMI), the Chi-square test or Fisher's exact test for categorical variables (e.g., Sex), and the Kruskal-Wallis test followed by Dunn's test with Bonferroni adjustment for ordered categorical variables (e.g., pain scores).

The associations between microbial taxa and inflammatory markers were evaluated in two steps to rigorously control for potential confounding effects. First, unadjusted correlations were assessed using Spearman's rank correlation. P-values from all microbial-marker pairs were adjusted for multiple comparisons using the Benjamini-Hochberg false discovery rate (FDR) method, with an adjusted q-value < 0.05 considered statistically significant.

Second, to evaluate whether these significant associations were independent of the confounding effects of diabetes mellitus (DM) hypertension (HBP), age and sex, we performed partial Spearman's correlation analyses adjusting for these two comorbidities. This analysis was implemented in Python using the rank-residual method: both the microbial abundance and inflammatory marker data were rank-transformed, and their residuals after linear regression against the ranks of DM, HBP, age, and sex were calculated. Age was included as a continuous variable, and sex was coded as male=1 and female=0. The Pearson correlation between these two sets of residuals constitutes the partial Spearman's correlation coefficient. The degrees of freedom for significance testing were calculated as df = n - k - 2, where k represents the number of covariates (k=4).The p-values from all partial correlation tests were similarly subjected to FDR correction across all microbe-marker pairs. Missing data were handled using pairwise deletion in all correlation analyses.

A *post hoc* power analysis was performed using G*Power 3.1 (Faul et al., 2009) to assess the statistical power of the study given the achieved sample size. The analysis was configured for a one-way ANOVA test, with an α level of 0.05.

### 2.5 Bioinformatic and microbiome community analysis

Raw sequencing reads were processed for quality control using Trimmomatic (v0.39) (Bolger et al., 2014) with the following parameters: ILLUMINACLIP : TruSeq3-SE.fa:2:30:10 LEADING:3 TRAILING:3 SLIDINGWINDOW:4:15 MINLEN:36. This pipeline performed adapter trimming using the Illumina TruSeq3-SE adapter sequences, removed leading and trailing low-quality bases (quality score < 3), and applied a sliding window approach that trimmed reads when the average quality score in a 4-base window fell below 15 (Q15). Reads shorter than 36 base pairs after trimming were discarded to ensure only high-quality sequences of sufficient length were retained for subsequent analysis. The resulting high-quality, non-redundant reads were aligned to the human reference genome (GRCh38/hg38) (Nassar et al., 2023) using the Burrows-Wheeler Aligner (BWA) (Li and Durbin, 2010, Li and Durbin, 2009) to subtract host-derived sequences.

For taxonomic classification, the remaining non-human reads were aligned against the NCBI nucleotide (nt) database using BLASTN (Altschul et al., 1990) with the "taxonomy" mode enabled. The lowest common ancestor (LCA) (Gori et al., 2011) algorithm was used to determine the final taxonomic assignment for each read. Reads assigned to the kingdoms Viridiplantae (plants) or Metazoa (animals, other than human) were excluded from further analysis to minimize environmental or dietary contamination. The proportion of reads assigned to these kingdoms was quantified and found to be minimal (averaging less than 1.1% combined) across all samples, confirming that their exclusion did not substantially impact the subsequent microbial community analysis. A species-level abundance table was then generated by summarizing the taxonomic assignments.

The final abundance table was imported into R (v4.2.3) (R Core Team, 2020) for downstream ecological and statistical analyses. Alpha diversity indices (Chao1, Observed species, Shannon, Simpson, and Pielou’s evenness) were calculated to assess within-sample diversity. Beta diversity, representing inter-sample dissimilarity, was computed using Bray-Curtis distances and visualized with principal coordinates analysis (PCoA). To test for significant differences in overall microbial community structure between severity groups, a permutational multivariate analysis of variance (PERMANOVA) was performed using the adonis2 function from the vegan package with 9999 permutations. This global test assesses the overarching hypothesis of whether community composition differs among groups, and its permutation-based p-value inherently controls the Type I error rate for this specific comparison. Following a significant global test, *post-hoc* pairwise PERMANOVA comparisons were conducted to delineate differences between specific severity groups (mild vs. moderate, mild vs. severe, moderate vs. severe). To control the false discovery rate across these multiple pairwise tests, p-values were adjusted using the Benjamini-Hochberg method.

To identify microbial profiles associated with disease severity, two approaches were used. First, differentially abundant taxa were identified using the Linear Discriminant Analysis Effect Size (LEfSe) method. All identified taxa were ranked by the absolute value of their Linear Discriminant Analysis (LDA) score. To focus the biological interpretation on the most salient biomarkers, the analysis was limited to the top 100 ranked features, which corresponded to an LDA score threshold of > 3.76.

Second, a Random Forest model, implemented with the randomForest package using 10,000 trees, was employed as a feature selection tool to rank the importance of microbial features in distinguishing infection severity. The model's performance was robustly assessed using 10-fold cross-validation. This approach allowed us to identify the most influential microbial taxa that correlate with infection severity, which were then prioritized for further analysis.

## 3 Result

### 3.1 Clinical characteristics and inflammatory profiles correlate with infection severity

The study cohort comprised 197 patients stratified into mild (n = 90), moderate (n = 41), and severe (n = 66) infection groups (**Figure 1**). Baseline demographics, including age, BMI, and sex distribution, did not differ significantly among the three groups. However, key clinical indicators of severity showed a clear association with the grading. The incidence of fever, dyspnea, and dysphagia was significantly elevated in the moderate and severe groups compared to the mild group (p<0.05 for all). Similarly, patient-reported pain intensity increased with severity, with the proportion of patients reporting the most severe pain (score 3) rising from 22.2% in the mild group to 50.0% in the severe group (p < 0.001). The incidence of Trismus also increased significantly with disease severity, rising from 37.8% in the mild group to 72.7% in the severe group (p < 0.001). The prevalence of comorbidities like diabetes and hypertension also generally increased with severity, with diabetes prevalence peaking in the severe group and hypertension in the moderate group (**Table 1**).

**Table 1:** Demographics and clinical characteristics of the study cohort.

Characteristics	Mild(n=90)	Moderate(n=41)	Severe(n=66)	P value
Age (years)	52.42 ± 16.89	58.07 ± 15.83	54.93 ± 16.4	0.126
BMI	24.81 ± 5.80	25.12 ± 5.22	23.79 ± 4.51	0.366
Male	62 (68.9%)	28 (68.3%)	44 (66.7%)	0.957
Clinical symptoms				
Fever, n%	22 (24.7%)	17 (40.5%)	29 (44.6%)	0.025
Dyspnea	18 (20%)	29 (70.7%)	42 (63.6%)	P<0.001
Dysphagia	40(44.4%)	29(70.7%)	56(84.8%)	P<0.001
Trismus	34(37.8%)	27(65.9%)	48(72.7%)	P<0.001
Pain				
0	3 (3.3%)	5 (12.2%)	4 (6.1%)	P<0.001
1	48 (53.3%)	8 (19.5%)	14 (21.2%)	
2	19 (21.1%)	12 (29.3%)	15 (22.7%)	
3	20 (22.2%)	16 (39.0%)	33 (50.0%)	
Underlying disease				
DM	10 (11.1%)	16 (39.0%)	26 (39.4%)	P<0.001
HBP	20 (22.0%)	17 (41.5%)	60 (30.5%)	0.046
Cardiopathy	5 (5.6%)	2 (4.9%)	10 (5.1%)	0.964

Values are presented as mean ± standard deviation or n (%). P-values were calculated to assess differences across the three severity groups. For continuous variables (Age, BMI), a one-way ANOVA was used. For nominal categorical variables (Male, Fever, Dyspnea, Dysphagia, Trismus, DM, HBP, Cardiopathy), the Chi-square test was used. For ordered categorical variables (Pain), the Kruskal-Wallis H test was used. The Pain score was graded on a scale from 0 to 3. BMI, Body Mass Index; DM, Diabetes Mellitus; HBP, High Blood Pressure.

**Figure 2:**
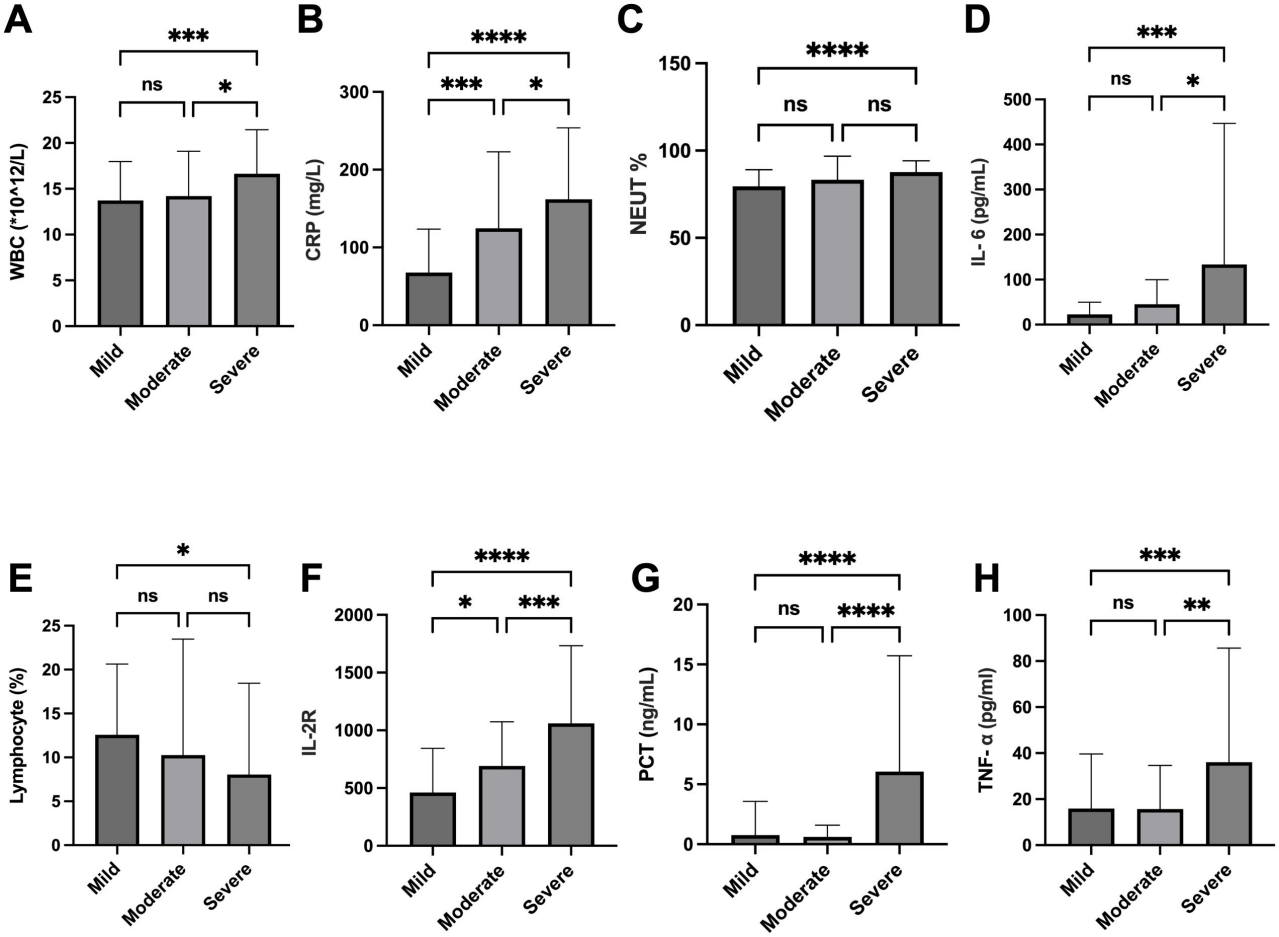
Analysis of blood routine between mild (n = 90), moderate (n = 41), and severe (n = 66) groups. **(A)** Statistical analysis of white blood cell count (WBC). **(B)** Statistical analysis of C-reactive protein (CRP). **(C)** Statistical analysis of neutrophil percentage (NEUT%). **(D)** Statistical analysis of interleukin-6 (IL-6). **(E)** Statistical analysis of lymphocyte percentage. **(F)** Statistical analysis of IL-2 receptor. **(G)** Statistical analysis of procalcitonin (PCT). **(H)** Statistical analysis of tumor necrosis factor-alpha (TNF-α). *p < 0.05, **p < 0.01, ***p < 0.001, ****p < 0.0001; ns: not significant.

To validate our severity stratification at a biological level, we analyzed routine blood parameters and a panel of inflammatory cytokines (**Figure 2**; **Supplementary Figure 1**). Several markers exhibited a clear, stepwise increase with infection severity. Specifically, the severe group showed significantly higher levels of WBC (**Figure 2A**), CRP (**Figure 2C**), IL-1β (**Supplementary Figure 1A**), IL-8 (**Supplementary Figure 1B**), and sIL-2R (**Supplementary Figure 1C**) when compared to both the mild and moderate groups (p < 0.05 for all comparisons).Other markers displayed a more nuanced pattern. PCT (**Figure 2E**), TNF-α (**Figure 2D**), and IL-6 (**Figure 2F**) were also highest in the severe group, but this elevation was only statistically significant relative to the moderate group. In contrast, the NEUT<ns/> (**Figure 2B**) was significantly lower in the mild group compared to both the moderate and severe groups, which did not differ from one another. Correspondingly, the LYMPH% (**Supplementary Figure 1D**) was significantly lower in the severe group than in the mild group, consistent with severe infection-induced lymphopenia.

To quantitatively assess the robustness of these observed differences, a *post hoc* power analysis was conducted. Based on the pronounced inter-group differences in CRP levels, a central inflammatory marker in this study, the calculated effect size was large (Cohen's f = 0.534). Given this effect and the total sample size of 197, the achieved statistical power exceeded 0.99, confirming that the study was highly sufficiently powered to detect the clinically relevant differences across the severity spectrum.

Collectively, these findings delineate a stepwise, escalating inflammatory gradient that tracks with clinical severity from mild to severe.

### 3.2 Microbial community structure shifts with infection severity

**Figure 3:**
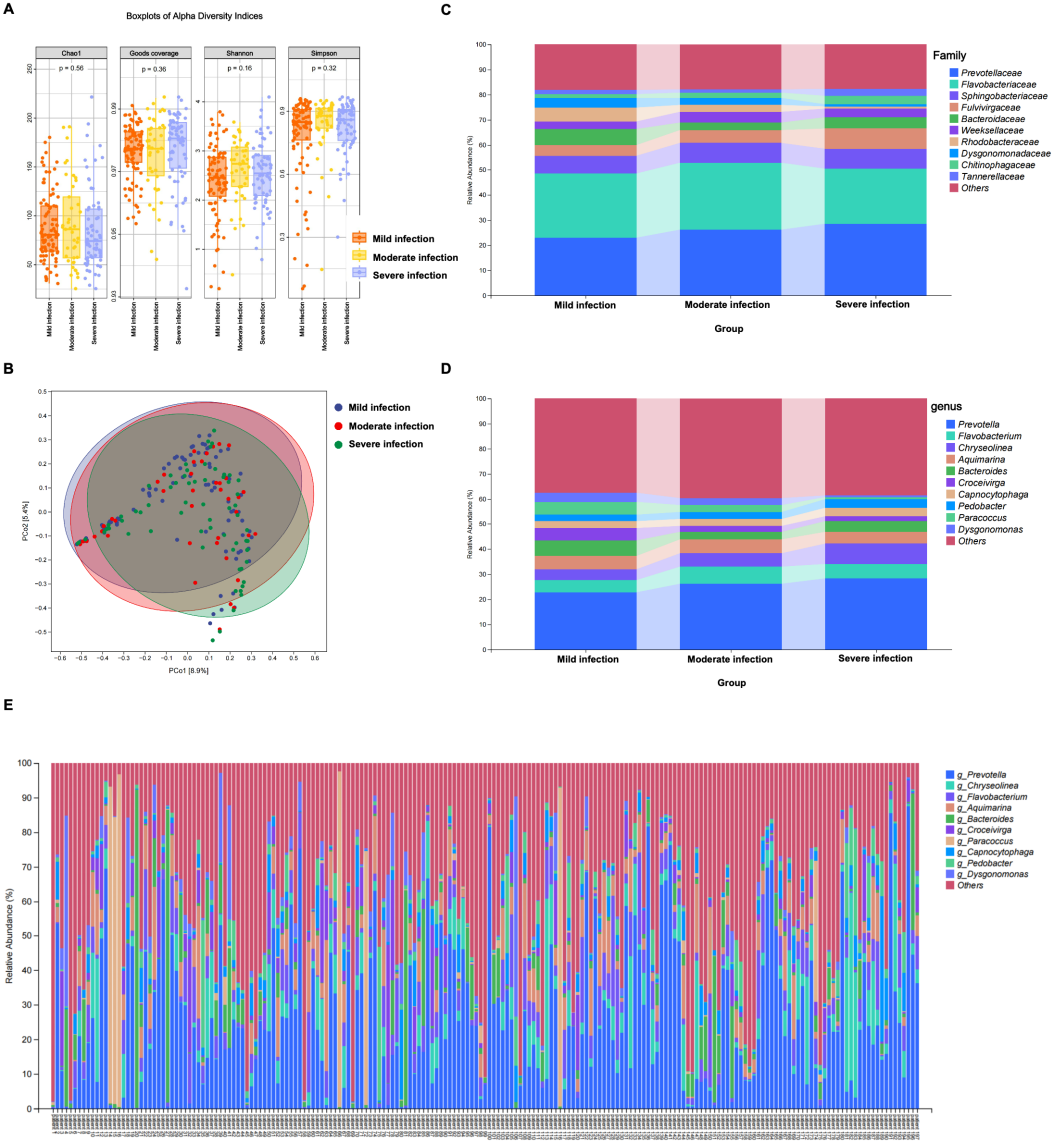
Microbial community structure and diversity across infection severity groups. **(A)** Boxplots of alpha diversity indices (Chao1, Goods coverage, Shannon, Simpson) across mild, moderate, and severe infection groups. No significant differences in diversity were observed between the groups for any of the indices (p > 0.05). **(B)** Principal coordinate analysis (PCoA) of Bray-Curtis metrics. (PERMANOVA, p < 0.05) **(C)** Stacked bar plot of the microbial community composition at the family level **(D)** Stacked bar plot of the microbial community composition at the genus level. **(E)** Stacked bar plot of the microbial community composition at the genus level for each individual pus sample (N = 197). Each bar represents one sample. The colored segments indicate the relative abundance of the top genera, while the 'Others' (in red) segment aggregates all remaining low-abundance genera (none of which met the predominance threshold). This panel visually highlights the high degree of heterogeneity and the variable predominance pattern observed among individual patients.

To investigate changes in microbial community structure across different infection severity levels, we first assessed α-diversity and β-diversity among samples from mild, moderate, and severe infection groups. Analysis of α-diversity revealed that while bacterial richness and evenness varied across groups, no significant differences were observed in commonly used diversity metrics, including the Chao1 index for species richness (p = 0.56), Shannon’s diversity index for overall species diversity (p = 0.16), Simpson’s diversity index (p = 0.32), and Good’s coverage index (p = 0.36) (**Figure 3A**). These results suggest that infection severity does not significantly alter the overall complexity of microbial communities within individual samples. In contrast, β-diversity analysis revealed significant differences in microbial community composition between severity groups. Principal coordinate analysis (PCoA) based on Bray-Curtis dissimilarities revealed a significant separation in microbial community composition between the severity groups (PERMANOVA, p < 0.05). Notably, *post-hoc* pairwise comparisons indicated that the moderate group did not show significant separation from either the mild or severe groups, suggesting it may represent a transitional state in the microbial succession. While some overlap was observed, the samples generally clustered according to infection severity, with the moderate group exhibiting an intermediate composition that overlapped with both mild and severe groups (**Figure 3B**). *Post-hoc* pairwise PERMANOVA tests confirmed a significant divergence specifically between the mild and severe groups (p-adjusted = 0.001), while the moderate group did not differ significantly from either. Thus, while overall microbial diversity remains similar across groups, the specific genera constituting these communities undergo a threshold-like shift rather than a linear progression, with a major restructuring occurring between mild and severe infections.

Furthermore, to further explore the commonalities and uniqueness of these microbial communities, we performed a Venn diagram analysis (**Supplementary Figure 2**). Among the total 5613 bacterial genera, 2,021 were shared across all three groups, representing the core microbiota. The number of genera unique to the severe infection group (1,326) was significantly higher than those in the mild (652) and moderate (415) infection groups. This suggests that as infection severity increases, there may be a shift in the dominant bacterial genera, as well as the colonization or expansion of several new genera that are rarely found in mild or moderate infection environments.

To elucidate the taxonomic features associated with infection severity, we performed taxonomic and relative abundance analysis at the family and genus levels based on microbial community composition in pus samples from each group. As shown in **Figure 3C**, at the family level, the microbial communities in pus were primarily dominated by *Prevotellaceae* and *Flavobacteriaceae*. The relative abundance of *Prevotellaceae*, *Fulvivirgaceae*, *Chitinophagaceae*, and *Tannerellaceae* gradually increased from the mild infection group to the severe infection group. In contrast, the relative abundance of *Bacteroidaceae*, *Rhodobacteraceae*, and *Dysgonomonadaceae* was highest in the mild infection group and decreased progressively with increasing infection severity. The relative abundance of *Sphingobacteriacea*e was similar across all three groups.

At the genus level (**Figure 3D**), this succession pattern becomes more pronounced. The relative abundance of *Prevotella* and *Chryseolinea* gradually increased from the mildly infected group to the severely infected group, becoming the dominant genera in the severe infection group. In sharp contrast, the genera *Bacteroides*, *Croceivirga*, *Paracoccus*, and *Dysgonomonas*, which were abundant in the mildly infected group, showed a progressive decrease in abundance as infection severity increased.

To investigate the individual-level variability and provide a clinically oriented perspective on potential causative agents, we visualized the microbial composition of all 197 pus samples at the genus level (**Figure 3E**), which vividly illustrates the highly heterogeneous nature of the infections. We subsequently identified the predominant genus (relative abundance ≥ 30%) within each sample, revealing a landscape of considerable etiological heterogeneity (**Table 2**). *Prevotella* was the most frequently identified predominant genus, observed in 59 (29.9%) samples. However, no single genus dominated the majority of cases, with other genera such as *Chryseolinea* (8 samples, 4.1%), *Bacteroides* (6 samples, 3.0%), and *Paracoccus* (6 samples, 3.0%) being dominant in smaller subsets.

**Table 2:** Frequency of predominant genera in OMSI pus samples (N = 197).

Predominant Genus (Relative Abundance > 30%))	Frequency	Percentage of Total Samples (%)
*Prevotella*	59	28.2%
*Chryseolinea*	8	3.8%
*Flavobacterium*	1	0.5%
*Aquimarina*	2	1.0%
*Bacteroides*	6	2.9%
*Croceivirga*	3	1.4%
*Paracoccus*	6	2.9%
*Capnocytophaga*	1	0.5%
*Pedobacter*	3	1.4%
*Dysgonomonas*	3	1.4%
Summary statistics		
Total samples with a predominant genus	92	46.7%
Total samples with no predominant Genus(<30%)	105	53.3%
Total Samples Analyzed	197	100%

A predominant genus was defined as any genus constituting ≥30% of the relative abundance in a single sample. Among the over 1700 genera detected, only the 10 listed in this table ever met this predominance criterion in at least one sample. The 'Frequency' column indicates the number of individual samples in which that specific genus was found to be predominant. The total count of samples with a predominant genus is 92 (out of 197 total samples). 105 samples (53.3%) were found to be highly polymicrobial, with no single genus reaching the 30% threshold. OMSI, oral and maxillofacial space infection; N, total number of samples.

Analysis of the predominance pattern revealed two distinct microbial community structures: first, 92 samples (46.7%) contained a single predominant genus that met the ≥30% threshold; second, the remaining 105 samples (53.3%) exhibited a highly polymicrobial structure where no single genus reached this threshold, as visualized by the large 'Others' segment in **Figure 3E**.

To provide clinically relevant resolution at the species level, we further analyzed the 92 samples with genus-level predominance (**Table 2**) to identify specific bacterial species exceeding the 30% relative abundance threshold (**Supplementary Table S3**). Among the 59 samples with *Prevotella* predominance, several species were identified as the dominant taxon, including *Prevotella veroralis* and *Prevotella scopos*, with no single species dominating across all cases. Notably, examination of all 92 samples revealed that not a single case was dominated by classic abscess-forming pathogens from the *Streptococcus anginosus* group (*S. anginosus*, *S. constellatus*, *S. intermedius*) or other well-known pathogens such as Streptococcus pyogenes. These findings underscore the polymicrobial and patient-specific nature of OMSI etiology, challenging the notion of predictable, single-pathogen infections.

These findings collectively indicate considerable variability in causative agent profiles among individual OMSI patients, with roughly half of the infections characterized by a clear predominant genus and the other half by a highly mixed, multi-taxa community.

### 3.3 LEfSe and Random Forest analyses identify robust microbial profiles of disease severity

To identify microbial groups capable of distinguishing stages of infection severity, we employed two independent, complementary approaches: Linear Discriminant Analysis Effect Size (LEfSe) and a Random Forest (RF) machine-learning model.

**Figure 4:**
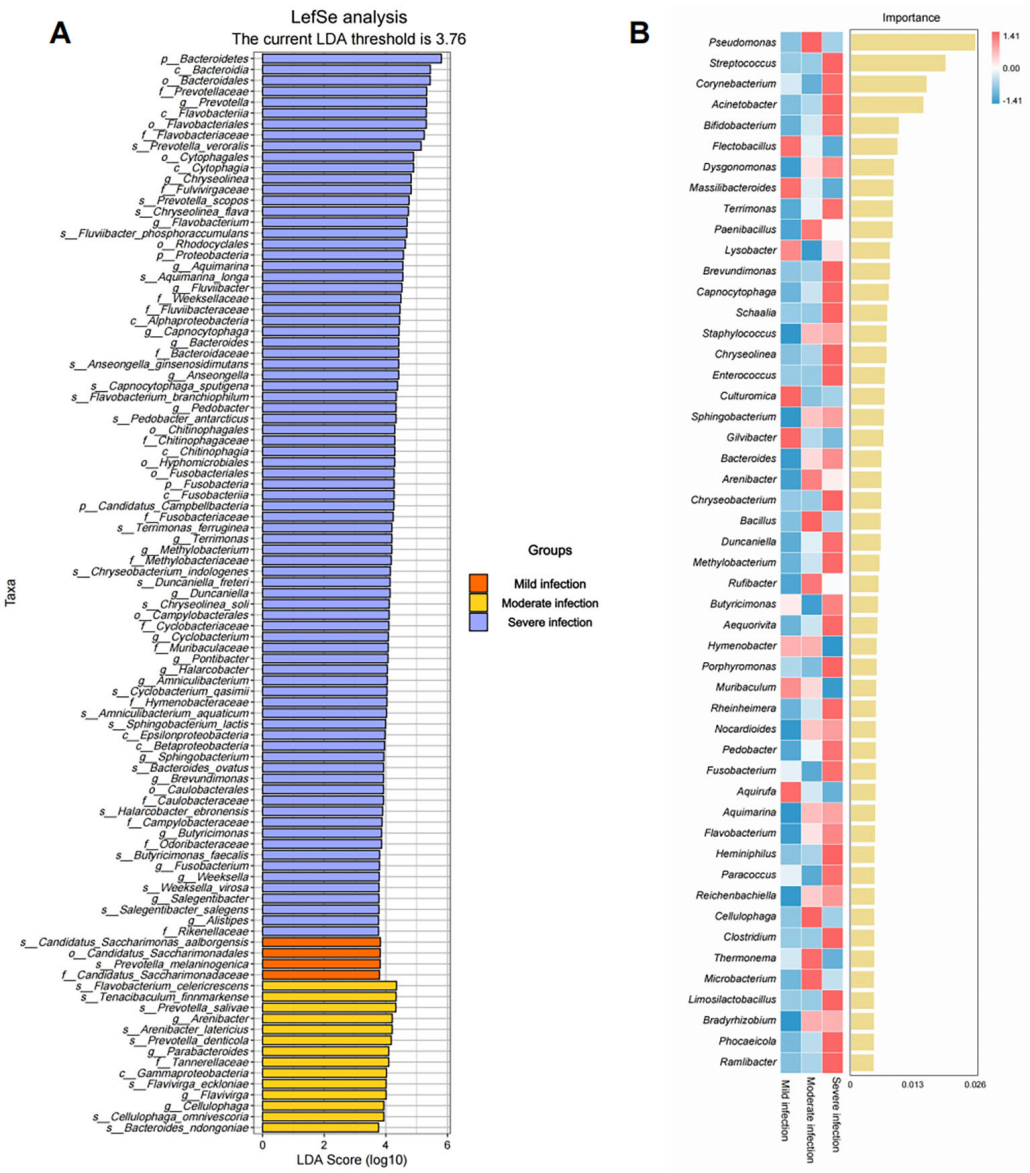
LEfSe analysis and random forest identify key microbial genera associated with infection severity. **(A)** Linear Discriminant Analysis Effect Size (LEfSe) analysis showing the taxa that are differentially abundant across the mild (orange), moderate (yellow), and severe (blue) infection groups. The LDA threshold of 3.76 is indicated by the dashed line. The top 100 most differentially abundant microbial taxa identified by LEfSe analysis using an LDA threshold of >3.76. This threshold was selected to prioritize the taxa with the strongest discriminatory ability between infection severity groups. **(B)** Random Forest feature importance analysis showing the importance of different genera for distinguishing infection severity.

First, LEfSe identified features showing significant differences across taxonomic ranks (LDA score > 3.76). These differential features are ranked by effect size in **Figure 4A**, and their taxonomic relationships are visualized in a cladogram in **Supplementary Figure 3**. At the phylum level, enrichment of Bacteroidetes was the most prominent feature of the severe infection group. At the genus level, 23 genera—including *Prevotella*, *Chryseolinea*, and *Flavobacterium*—exhibited elevated LDA scores. At the species level, *Prevotella veroralis*, *Prevotella scopos*, and *Chryseolinea flava* emerged as key discriminatory taxa of severe infection. In stark contrast, the moderate infection group was characterized by significantly higher abundances of four genera, including *Arenibacter*, *Parabacteroides*, *Flavivirga and Cellulophaga*, relative to the other groups. The mild infection group showed significant enrichment of *Candidatus* Saccharimonas aalborensis and *Prevotella melaninogenica*.

Next, to validate and rank the importance of these differential taxa, we constructed an RF model as a feature selection tool. The model successfully discriminated samples across severity groups and produced per-genus importance scores (**Figure 4B**). The top-ranking genera were *Pseudomonas*, *Streptococcus*, *Corynebacterium*, *Acinetobacter*, *Bifidobacterium*, *Flectobacillus*, *Dysgonomonas*, *Massilibacteroides*, *Terrimonas*, and *Paenibacillus*. Notably, the relative abundances of *Streptococcus*, *Corynebacterium*, *Acinetobacter*, *Bifidobacterium*, *Dysgonomonas*, and *Terrimonas* increased with infection severity, whereas *Flectobacillus* and *Massilibacteroides* were most abundant in the mild group and decreased progressively in the moderate and severe groups, consistent with depletion along the disease course.

Given that Streptococcus is both a dominant constituent of the oral microbiota (Mclean et al., 2022; Abranches et al., 2018) and a top-ranking predictor in our genus-level Random Forest model, we performed a follow-up investigation at the species level to determine if a specific species was driving this signal. We constructed a new species-level Random Forest model and generated a correlation heatmap between species and clinical inflammatory markers. Interestingly, these higher-resolution analyses did not identify any single Streptococcus species as a top-ranking discriminatory feature. The most important species for predicting severity were consistently from other genera, such as *Sphingobacterium compositi*, *Flavobacterium album*, and *Brevundimonas intermedia*, corroborating our genus-level findings (**Supplementary Figure 4A**). Similarly, the correlation heatmap did not reveal a specific *Streptococcus* species with a uniquely strong correlation pattern with key inflammatory markers. (**Supplementary Figure 4B**). Species-level analysis was conducted to investigate the potential role of classic abscess-forming streptococci (e.g., the *S. anginosus* group). Notably, species such as *S. anginosus*, *S. constellatus*, and *S. intermedius* were either not detected or were present only at extremely low relative abundances (e.g., < 3 × 10^-8^) in severe infections (**Supplementary Table S2**). This indicates that the pathogenicity of the *Streptococcus* genus in our OMSI cohort is not driven by these canonical pathogens but is more likely a collective effect of the genus within a polymicrobial consortium.

These results suggest that while the genus *Streptococcus* is an important component of the pathogenic community, its association with severity is likely a collective, genus-wide effect or a result of synergistic interactions, rather than being driven by a single dominant species within the genus.

### 3.4 Correlation of microbial profiles with host inflammatory status

**Figure 5:**
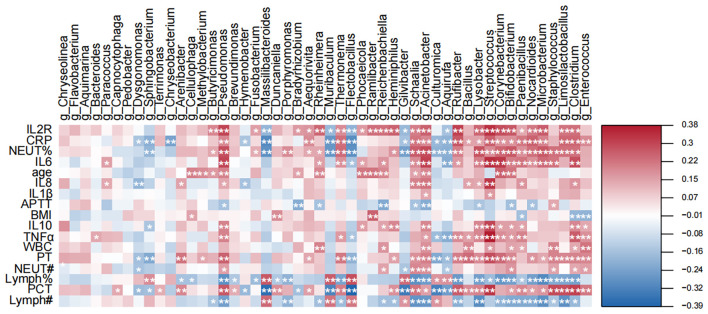
Correlation analysis reveals associations between key microbial genera and host inflammatory status. This heatmap displays the Spearman correlation coefficients between the relative abundances of the most predictive bacterial genera (identified by Random Forest analysis) and a comprehensive panel of host clinical and laboratory markers. The intensity of the color corresponds to the strength of the correlation (red for positive, blue for negative). Only statistically significant correlations are marked with an asterisk (*q < 0.05; ** q < 0.01).

To further interrogate relationships between the pus microbiome and host pathophysiology, we performed Spearman’s rank correlation to evaluate associations between bacterial genera identified by the random forest (RF) model and clinical/laboratory indicators (**Figure 5**). This analysis delineated two microbial consortia with opposing correlation patterns to host inflammatory status. One group, which we termed a “consortium associated with a pro-inflammatory state,” was represented by *Streptococcus*, *Corynebacterium*, *Acinetobacter*, and *Bifidobacterium*. The abundances of these genera, particularly the robustly significant *Streptococcus*, *Acinetobacter*, and *Bifidobacterium*, showed significant positive correlations (FDR-adjusted *q* < 0.05). with core systemic inflammatory markers, including CRP, WBC, NEUT% and PCT. These genera also correlated positively with cytokines such as IL-6 and sIL-2R, consistent with expansion that is concurrent with host immune activation. Notably, *Streptococcus*, *Corynebacterium*, *Acinetobacter*, and *Bifidobacterium* exhibited increasing abundance with worsening infection severity in the RF analysis.

In contrast, another set of taxa like *Flectobacillus*, *Muribaculum*, and *Massilibacteroides*, which we identified as a “group associated with an anti-inflammatory profile”, displayed strong and statistically robust significant negative correlations with the inflammatory markers, but significant positive correlations with both lymphocyte percentage and absolute lymphocyte count (FDR-adjusted *q* < 0.05). Consistently, these genera were most abundant in the mild infection group in the RF analysis and declined with increasing severity, suggesting that their depletion may mark disease progression and a shift toward heightened systemic inflammation.

To rigorously assess whether these microbiome-inflammation relationships were independent of potential confounding by comorbidities, age, and sex, we performed partial Spearman’s correlation analyses adjusting for diabetes mellitus (DM), hypertension (HBP), age, and sex. After this adjustment and FDR correction, several genera remained significantly associated. For instance, *Streptococcus* abundance was independently and positively correlated with both CRP (partial ρ = 0.301, FDR-corrected q = 0.0015) and IL−6 (partial ρ = 0.273, FDR-corrected q = 0.0050). Similarly, *Corynebacterium* showed significant independent correlations with CRP (partial ρ = 0.275, FDR-corrected q = 0.0046) and IL−6 (partial ρ = 0.259, FDR-corrected q = 0.0086). The association between *Acinetobacter* and CRP also remained significant (partial ρ = 0.331, *q* = 0.00039). Notably, the association between *Prevotella* and CRP was particularly strong and remained highly significant after full adjustment (partial ρ = 0.372, FDR-corrected *q* = 7.02e-05). It is worth noting that not all associations remained significant after this stringent adjustment; for example, the correlations of *Acinetobacter* and *Prevotella* with IL-6 were attenuated and did not survive FDR correction (both *q* > 0.13, see **Supplementary Materials**), suggesting that these specific relationships may be more susceptible to the influence of the included covariates. This confirms that the core relationships between the pus microbiome and the host systemic inflammatory response are robust and not solely driven by the confounding effects of these comorbidities.

## 4 Discussion

Oral and maxillofacial space infections (OMSI) have long been treated based on classical bacteriology, yet the drivers of disease severity remain poorly understood. This study, employing metagenomic next-generation sequencing (mNGS), moves beyond single-pathogen identification to reveal a more complex narrative: OMSI progression is underpinned by a dynamic microbial succession, where functionally distinct consortia appear to compete and shift in dominance, are directly associated with shifts in the host's systemic inflammatory response. These findings provide a novel ecological framework for understanding OMSI pathogenesis and lay the groundwork for targeted therapeutic strategies.

A pivotal observation of our study is that while overall microbial diversity (α-diversity) remains stable, the community structure (β-diversity) shifts significantly with increasing infection severity. This challenges the common paradigm that disease progression is always accompanied by a loss of diversity (Pragman et al., 2024; Choi et al., 2024) and indicates that the transition to severe infection is a process of community turnover rather than collapse. Pairwise PERMANOVA tests revealed a significant divergence only between the mild and severe groups (p-adjusted = 0.001), with no significant differences between adjacent severity stages. This non-linear progression is consistent with the concept of an ecological threshold, where the community undergoes marked restructuring upon reaching a severity tipping point a view supported by the increased genus-level heterogeneity observed in severe infections.

It is well-established that the causative agents of OMSI originate from the resident oral microbiota. A healthy oral cavity harbors a complex and largely symbiotic microbial community, typically dominated by facultative anaerobic, Gram-positive bacteria such as *Streptococcus* and *Actinomyces*, as well as other commensal genera like *Veillonella* and *Neisseria* (Zhang et al., 2018; Zaura et al., 2009; Mark Welch et al., 2020). This balanced ecosystem, maintained by host factors and microbial interactions, is crucial for oral health (Freire et al., 2021; Radaic and Kapila, 2021). However, shifts in environmental conditions, such as poor oral hygiene, dental procedures, or compromised host immunity can disrupt this balance, allowing opportunistic pathogens or pathobionts within the community to proliferate and initiate infection (Cornejo Ulloa et al., 2019). This transition from a commensal to a pathogenic state is evident in the specific taxa identified in our data. In mild infections, the microbiome is characterized by taxa associated with a relatively balanced oral environment, such as *Candidatus Saccharimonas* and *Prevotella melaninogenica* (Guo et al., 2024; Könönen and Gursoy, 2021; Solch-Ottaiano et al., 2024; Xie et al., 2021). As the disease progresses, these communities give way to consortia dominated by potent biomarkers of severe disease. Notably, LEfSe analysis identified anaerobic genera, particularly *Prevotella*, as the most significant single indicator of severe infection. This aligns with its known role as a key player in the hypoxic and nutrient-rich abscess microenvironment (Darveau, 2010), and its established involvement in oral diseases (Mättö et al., 1997; Könönen et al., 2022), localized infections (Brook and Frazier, 1998; Boyanova et al., 2010; Tsai et al., 2018; Mehmood et al., 2014) and systemic infections (Lu et al., 2023), driven by virulence factors like lipopolysaccharides (LPS) and proteolytic enzymes that mediate tissue destruction (Hitch et al., 2022). The enrichment of opportunistic genera like *Chryseolinea* and *Flavobacterium* in severe cases further suggests a synergistic pathogenic process under compromised host conditions (Kim et al., 2013; Benjamin et al., 2025; Guo-Zhong and Xiaolei, 2010).

To identify biomarkers with the greatest clinical utility, we applied two complementary analytical strategies: LEfSe and random forest (RF). As an effect-size-oriented univariate method, LEfSe identified *Prevotella* as the genus with the strongest independent discriminatory power. By contrast, the RF classifier employed as a feature selection tool ranked *Pseudomonas*, *Streptococcus*, *Corynebacterium*, *Acinetobacter*, and *Bifidobacterium* among the top microbial taxa associated with infection severity. The two feature-selection approaches yielded partially different key taxa, particularly for *Prevotella*. In LEfSe, *Prevotella* achieved a very high LDA score, indicating that, as a single feature, it provides the most statistically robust and largest effect size for distinguishing severity groups. However, in the RF model, *Prevotella* did not appear among the highest-importance predictors. This discrepancy reflects methodological complementarity rather than conflict: LEfSe highlights the biological significance of individual taxa, whereas RF identifies key microbial taxa that contribute most to the differentiation of infection severity, making it a powerful tool for feature selection.

Another important consideration is the role of *Streptococcus*, a genus that constitutes a major proportion of the oral microbiota. Given its significant heterogeneity with different species groups associated with health or disease, a genus-level analysis is insufficient for clinical interpretation. Following this rationale, we conducted a deeper, species-level investigation. However, while the genus Streptococcus was highly abundant, no single *Streptococcus* species emerged as a top-ranking biomarker for infection severity in our models. This finding suggests that the pathogenicity in severe OMSI may not be driven by a single dominant species. Instead, it points toward a polymicrobial process where the collective functions of a dysbiotic community contribute to the disease state. Within this context, certain genera strongly associated with inflammatory markers in our study, such as *Prevotella*, may act as primary drivers, while highly abundant genera like *Streptococcus* could contribute by creating a supportive environment for the wider pathogenic consortium. The pathogenic signal, therefore, appears to be distributed across the community rather than being concentrated in one particular species.

To bridge our community-level findings with clinical practice, we analyzed the predominant genus (defined as >30% relative abundance) within each patient sample. This approach connects traditional culture-based views with the complex reality revealed by mNGS. Consistent with classical perspectives, the anaerobic genus *Prevotella* was the most frequently identified dominant pathogen, constituting the leading organism in 59 of 197 (29.9%) cases. This prevalence provides a data-driven rationale for ensuring empiric antibiotic regimens include robust anaerobic coverage. However, a key finding was the considerable etiological heterogeneity beyond this single genus. A long tail of 102 other genera were found to dominate in individual infections, and a notable 15 (7.6%) samples presented a "highly mixed" profile where no single genus reached the predominance threshold. This diversity, largely invisible to conventional culture, indicates that for the majority of patients, the 'causative agent' may not be a single, predictable bacterium but a patient-specific pathogenic consortium.

This study is consistent with, and substantially extends, prior culture-based research. While traditional methods have identified pathogens like *Prevotella* and *Streptococcus* spp. in OMSI (Riggio et al., 2007; Al-Nawas and Maeurer, 2007; Farmahan et al., 2015; Wang et al., 2022b), their utility is hampered by low sensitivity for anaerobic and fastidious organisms (Shi et al., 2023). Leveraging the unbiased power of mNGS, we not only achieved higher detection rates but also uncovered previously underreported taxa linked to specific severity strata. Building on prior work (Shi et al., 2023)we extend the evidence base by severity-stratified, community-level profiling of the pus microbiome and its associations with a comprehensive panel of systemic inflammatory markers. Prior studies established correlations between individual markers like WBC, CRP, and IL-6 and OMSI severity (Brajkovic et al., 2022; Kim and Lee, 2021; Xiaojie et al., 2021; Wang et al., 2024; Kamiński et al., 2022), but our work forges a direct link between these systemic responses and specific microbial profiles. By demonstrating that RF-identified genera correlate tightly with inflammatory markers, our findings provide strong evidence for an association between microbial dysbiosis and host pathophysiology. This crucial connection lays the groundwork for future functional studies aimed at uncovering the precise mechanistic links. By mapping severity-associated microbial features, we deepen understanding of infection progression and lay the groundwork for precision diagnostics and subsequent functional studies (e.g., meta transcriptomics, metabolomics). Clinically, the high sensitivity (Wang et al., 2022a) and rapid turnaround time of mNGS underscore its promise as a transformative tool for early, precise OMSI diagnosis (Zhang et al., 2022).

These findings provide important guidance for optimizing clinical management of OMSI across severity strata. Current standardized care typically comprises prompt surgical incision and drainage together with empiric broad-spectrum antibiotics. However, our results indicate that infections of differing severity are underpinned by functionally distinct microbial communities, suggesting that a one-size-fits-all empiric regimen may be suboptimal. In mild-to-moderate infections, where communities enriched with genera like *Flectobacillus* and *Massilibacteroides* (which we found to be negatively correlated with inflammation) are prevalent, prompt surgical drainage combined with narrower-spectrum antibiotics targeting oral anaerobes (e.g., penicillin plus metronidazole) may be sufficient (Warnke et al., 2008). This approach could limit the overuse of broad-spectrum agents, reducing selection for resistance. In contrast, for severe infections characterized by the expansion of a true "pro-inflammatory microbiota", *Streptococcus*, *Corynebacterium*, and *Acinetobacter*, which showed strong positive correlations with CRP, PCT, and WBC, more aggressive therapy is warranted. For these patients, initial treatment should couple aggressive surgical drainage with rapid diagnostics (like mNGS) and empiric broad-spectrum regimens (e.g., β-lactams, quinolones) (Pang et al., 2019; Laborda et al., 2021) to ensure coverage of these potent inflammatory drivers, facilitating a swift transition to individualized therapy and potentially reducing life-threatening complications.

Despite its strengths, this study has several important limitations. First, limitations inherent to the study design may influence the generalizability and interpretation of our findings. The single-center design restricts the generalizability of our identified biomarkers, which, while robust within our cohort, require validation in larger, multicenter studies to confirm their universal applicability. The cross-sectional nature of the study allows us to establish strong associations but does not permit conclusions about causality. Furthermore, the retrospective design and our necessary exclusion criteria (e.g., no underlying malignancy or recent antibiotic use), while crucial for controlling confounders, introduce a potential selection bias, making our findings most applicable to non-compromised OMSI patients. Finally, we acknowledge the absence of a healthy control group. This design choice restricts our ability to definitively attribute the observed microbial profiles to the pathophysiology of OMSI itself, as compared to a healthy state. However, the primary objective of this research was not to distinguish diseased from healthy individuals, but rather to elucidate the pathophysiological continuum within a large, well-characterized cohort of OMSI patients. By comparing well-defined mild, moderate, and severe subgroups, our study robustly identifies key factors associated with disease progression, which holds considerable promise for developing biomarkers for severity assessment and prognosis.

Second, our analytical models have specific limitations. A critical point is the lack of an external validation cohort for our Random Forest model. Although its performance was robustly assessed using 10-fold cross-validation, this only ensures internal validity. Consequently, the model's generalizability and its utility as a standalone diagnostic tool remain unproven. Its primary role in this discovery-phase study was therefore to serve as a powerful feature-selection tool, corroborating findings from other analyses. Finally, practical challenges may hinder the immediate clinical translation of our findings. The current cost and complexity of mNGS are significant barriers to its widespread adoption in routine clinical practice. Therefore, future work is essential. This should include not only prospective, multicenter studies to validate our biomarkers and externally validate the predictive model, but also experimental work to elucidate causal relationships through functional studies (e.g., meta-transcriptomics, metabolomics). A key parallel goal should be the development of cost-effective, targeted diagnostic assays (e.g., qPCR panels) to translate these discoveries into accessible clinical tools.

In summary, this work provides the mNGS-based, severity-stratified analysis of microbiome dynamics in OMSI, offering a robust foundation for understanding pathogenesis, developing precision diagnostics, and optimizing therapeutic strategies. Prospective longitudinal studies that track microbiome trajectories and evaluate the clinical effectiveness of mNGS-guided interventions are warranted to determine their impact on patient outcomes.

## 5 Conclusion

In this study, we provide a comprehensive metagenomic analysis of the pus microbiome in patients with varying severities of oral and maxillofacial space infections. Our results demonstrate that while overall microbial richness does not significantly differ, the composition of the microbial community undergoes a statistically significant and clinically relevant shift with increasing infection severity.

A key conclusion of our work is the identification of two microbial consortia whose relative abundances are oppositely correlated with the host's inflammatory status. We identified a microbial profile headlined by genera such as *Streptococcus*, *Corynebacterium*, and *Acinetobacter*, which was associated with elevated systemic inflammatory markers (e.g., CRP, WBC, IL-6) and characteristic of severe infections. Conversely, another consortium, including *Flectobacillus* and *Massilibacteroides*, was linked to a healthier immune profile (higher lymphocyte counts) and was depleted as the infection worsened.

Furthermore, our analysis at the individual patient level revealed considerable etiological heterogeneity. While Prevotella was the most frequent dominant pathogen, a majority of cases were either dominated by a wide array of other, less common genera or presented as a highly mixed community with no single dominant organism. These findings suggest that the microbiome in OMSI is a dynamic ecosystem whose compositional shifts are associated with host pathophysiology. The microbial profile identified here, particularly the patient-specific dominant taxa, hold potential as biomarkers for assessing disease severity and may inform the development of targeted therapeutic strategies.

## Data Availability

The data presented in the study are deposited in the SRA (https://www.ncbi.nlm.nih.gov/sra/) repository, accession number PRJNA1379436.

## Supplementary material

Supplementary Figure 1 Comparison of inflammatory markers and lymphocyte percentage in the blood of patients with mild (N = 90), moderate (N = 41), and severe (N = 66) disease. **(A)** Statistical analysis of Interleukin-1 beta (IL-1β, pg/ml). **(B)** Statistical analysis of Interleukin-8 (IL-8, μg/L). **(C)** Statistical analysis of soluble IL-2 receptor (sIL-2R). (D) Statistical analysis of lymphocyte percentage (%).

Supplementary Figure 2 Microbial biomarkers distinguishing different infection severities. This LEfSe cladogram illustrates taxa that are significantly enriched in the Mild (orange), Moderate (yellow), or Severe (blue) infection groups. The hierarchy extends from phylum (inner) to species (outer) levels, and the node size reflects the relative abundance of the taxon.

Supplementary Figure 3 Venn diagram showing the distribution of bacterial genera among Mild, Moderate, and Severe infection groups. The numbers indicate the count of bacterial genera in each shared or unique section. A core of 2,021 genera was shared across all three groups. The number of genera unique to the Severe infection group (1,326) was notably higher than in the Mild (652) and Moderate (415) groups.

Supplementary Figure 4 Species-level analysis identifies key microbial features associated with infection severity**. (A)** Random Forest feature importance analysis showing the importance of different species for distinguishing infection severity. **(B)** Correlation analysis reveals associations between key microbial genera and host inflammatory status. This heatmap displays the Spearman correlation coefficients between the relative abundances of the most predictive species (identified by Random Forest analysis) and a comprehensive panel of host clinical and laboratory markers. The intensity of the color corresponds to the strength of the correlation (red for positive, blue for negative). Only statistically significant correlations are marked with an asterisk (*q < 0.05; ** q < 0.01).
