# Supplementary figures and images for: Metagenomic analysis reveals severity-dependent microbial succession and correlation with host inflammatory response in oral and maxillofacial space infections
Source: Front Cell Infect Microbiol. 2026 Jan 8;15:1695928. doi: 10.3389/fcimb.2025.1695928 (PMC12824008; doi:10.3389/fcimb.2025.1695928)

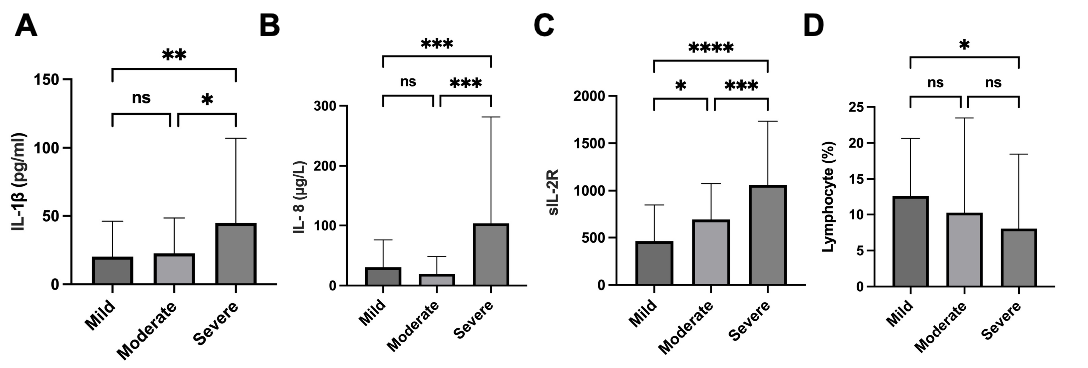

Supplement: Supplementary Figure 1 — Comparison of inflammatory markers and lymphocyte percentage in the blood of patients with mild (N = 90), moderate (N = 41), and severe (N = 66) disease. (A) Statistical analysis of Interleukin-1 beta (IL-1β, pg/ml). (B) Statistical analysis of Interleukin-8 (IL-8, μg/L). (C) Statistical analysis of soluble IL-2 receptor (sIL-2R). (D) Statistical analysis of lymphocyte percentage (%). [file Image1.tif]

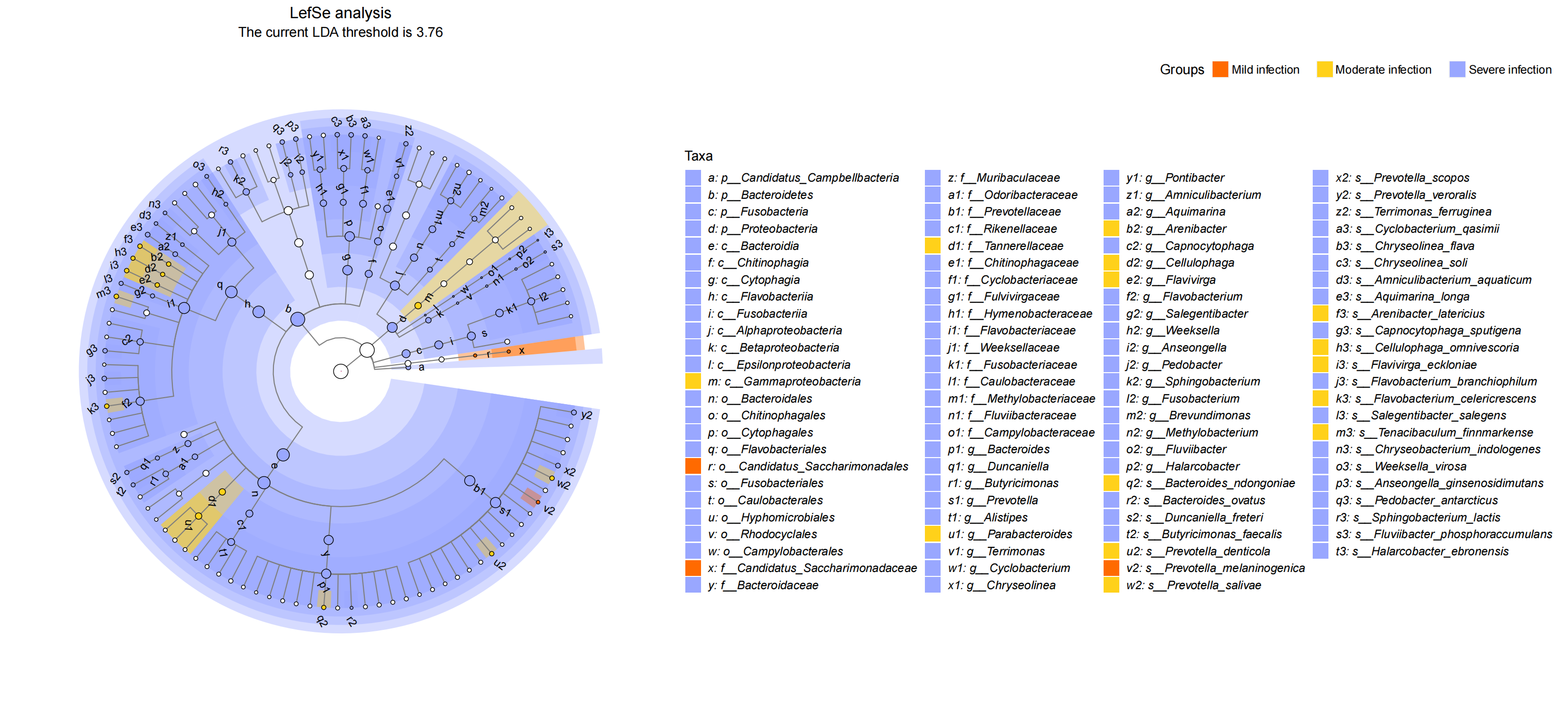

Supplement: Supplementary Figure 2 — Microbial biomarkers distinguishing different infection severities. This LEfSe cladogram illustrates taxa that are significantly enriched in the Mild (orange), Moderate (yellow), or Severe (blue) infection groups. The hierarchy extends from phylum (inner) to species (outer) levels, and the node size reflects the relative abundance of the taxon. [file Image2.tif]

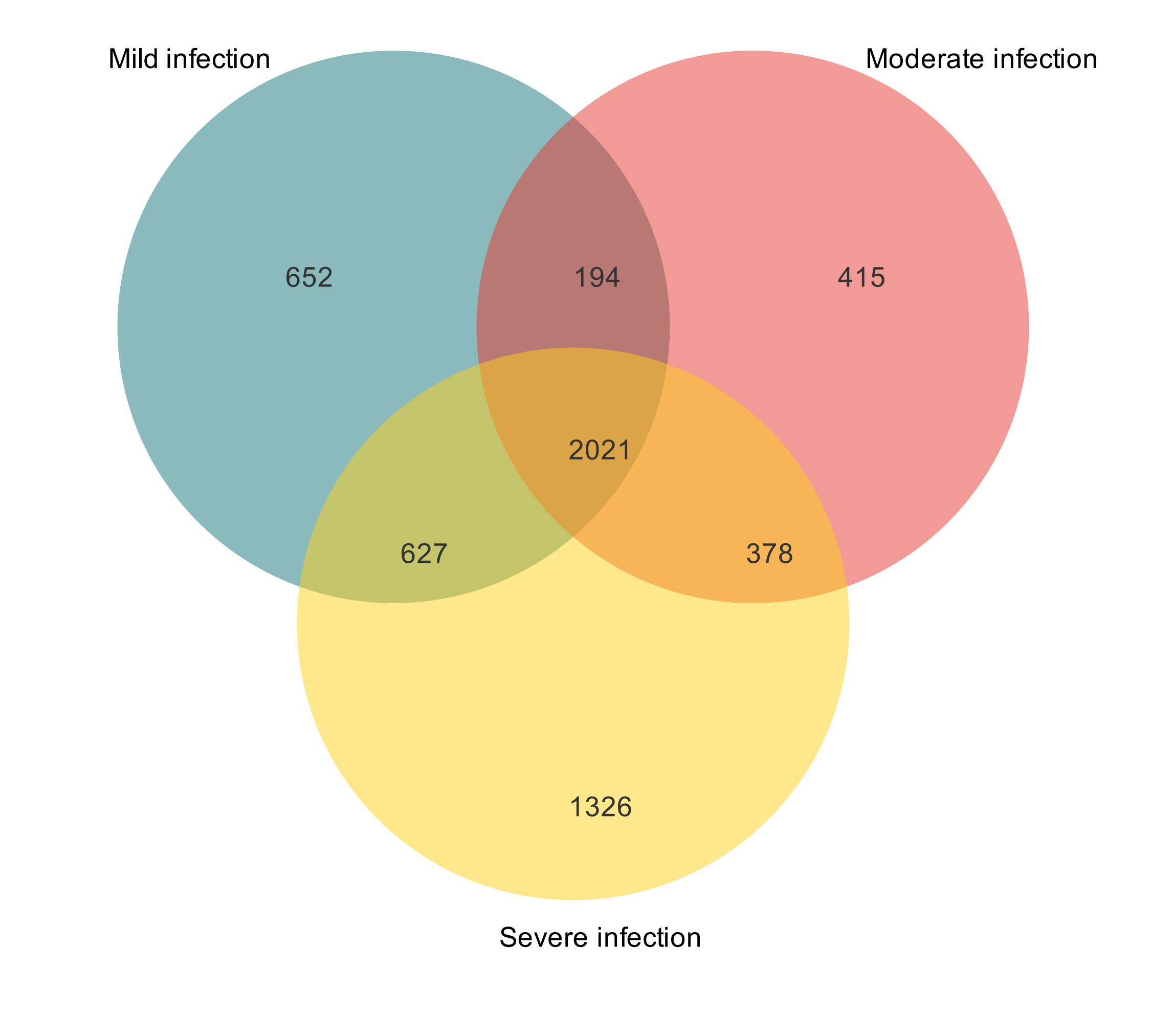

Supplement: Supplementary Figure 3 — Venn diagram showing the distribution of bacterial genera among Mild, Moderate, and Severe infection groups. The numbers indicate the count of bacterial genera in each shared or unique section. A core of 2,021 genera was shared across all three groups. The number of genera unique to the Severe infection group (1,326) was notably higher than in the Mild (652) and Moderate (415) groups. [file Image3.tif]

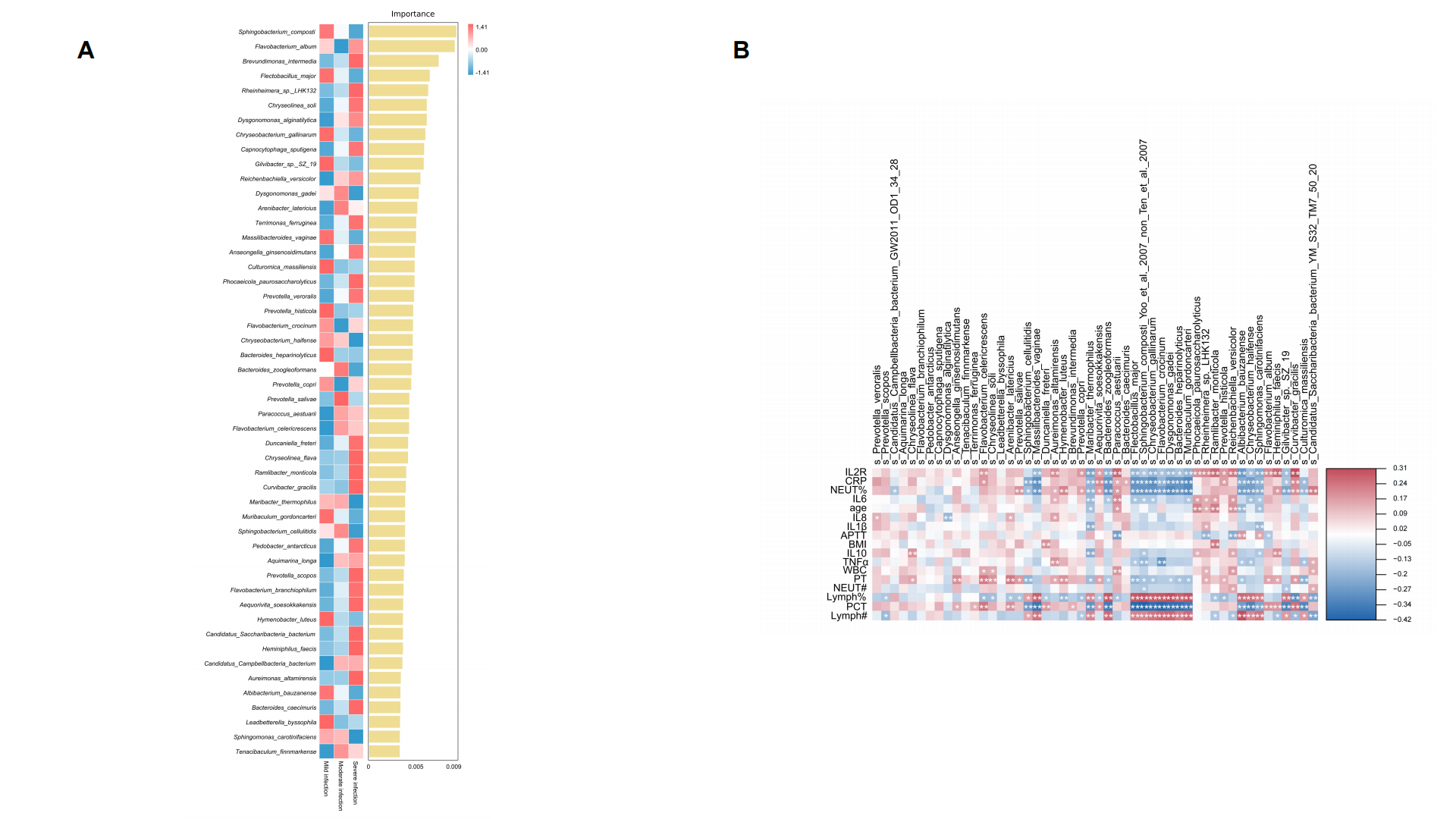

Supplement: Supplementary Figure 4 — Species-level analysis identifies key microbial features associated with infection severity. (A) Random Forest feature importance analysis showing the importance of different species for distinguishing infection severity. (B) Correlation analysis reveals associations between key microbial genera and host inflammatory status. This heatmap displays the Spearman correlation coefficients between the relative abundances of the most predictive species (identified by Random Forest analysis) and a comprehensive panel of host clinical and laboratory markers. The intensity of the color corresponds to the strength of the correlation (red for positive, blue for negative). Only statistically significant correlations are marked with an asterisk (*q < 0.05; ** q < 0.01). [file Image4.tif]
