# Supplementary material for: Metagenomic analysis reveals severity-dependent microbial succession and correlation with host inflammatory response in oral and maxillofacial space infections
Source: Front Cell Infect Microbiol. 2026 Jan 8;15:1695928. doi: 10.3389/fcimb.2025.1695928 (PMC12824008; doi:10.3389/fcimb.2025.1695928)
Supplement: Supplementary file 5 [file Table1.docx]

Supplementary Material

**Metagenomic analysis reveals severity-dependent microbial succession and correlation with host inflammatory response in Oral and maxillofacial space infections**

**Xijun Wang1,2,3,4,5 †, Lei Ye1,2,3,4,5 †, Yimin Liu1,2,3,4,5, Hui Li1,2,3,4,5, Huan Shi1,2,3,4,5*, Lingyan Zheng1,2,3,4,5***

^1^Department of Oral Surgery, Shanghai Ninth People's Hospital, Shanghai Jiao Tong University School of Medicine, Shanghai, China

^2^College of Stomatology, Shanghai Jiao Tong University, Shanghai, China

^3^National Center for Stomatology, Shanghai, China

^4^National Clinical Research Center for Oral Diseases, Shanghai, China

^5^Shanghai Key Laboratory of Stomatology, Shanghai, China

*** Correspondence:**

Corresponding Author: Lingyan Zheng,[zhenglingyan73@163.com](mailto:zhenglingyan73@163.com);Huan Shi, shihuan1312@163.com

†These authors contributed equally to this work and share first authorship

**Supplementary Figures**

**
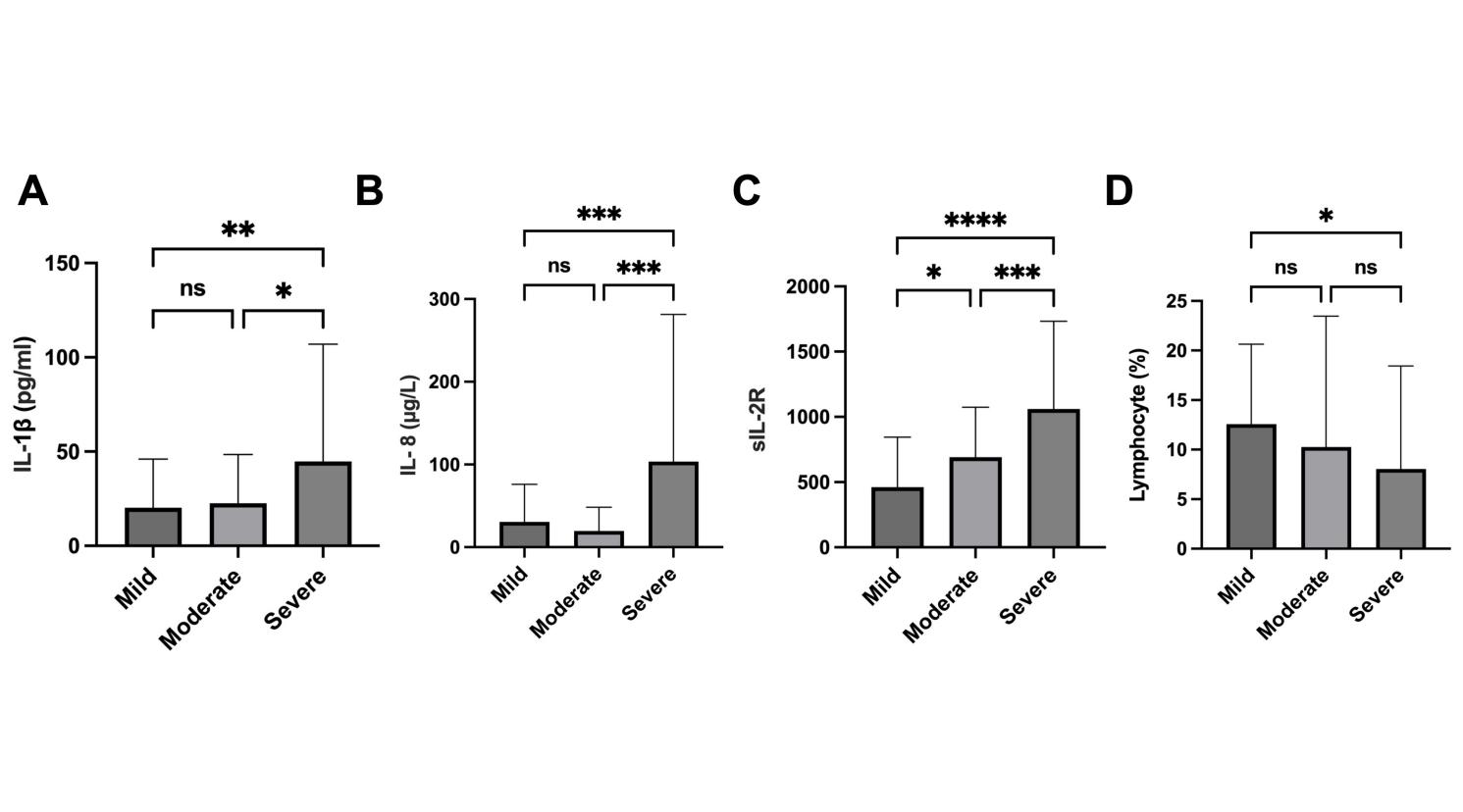
Supplementary Figure 1.** Comparison of inflammatory markers and lymphocyte percentage in the blood of patients with mild (N=90), moderate (N=41), and severe (N=66) disease. (A) Statistical analysis of Interleukin-1 beta (IL-1β, pg/ml). (B) Statistical analysis of Interleukin-8 (IL-8, μg/L). (C) Statistical analysis of soluble IL-2 receptor (sIL-2R). (D) Statistical analysis of lymphocyte percentage(%).

**
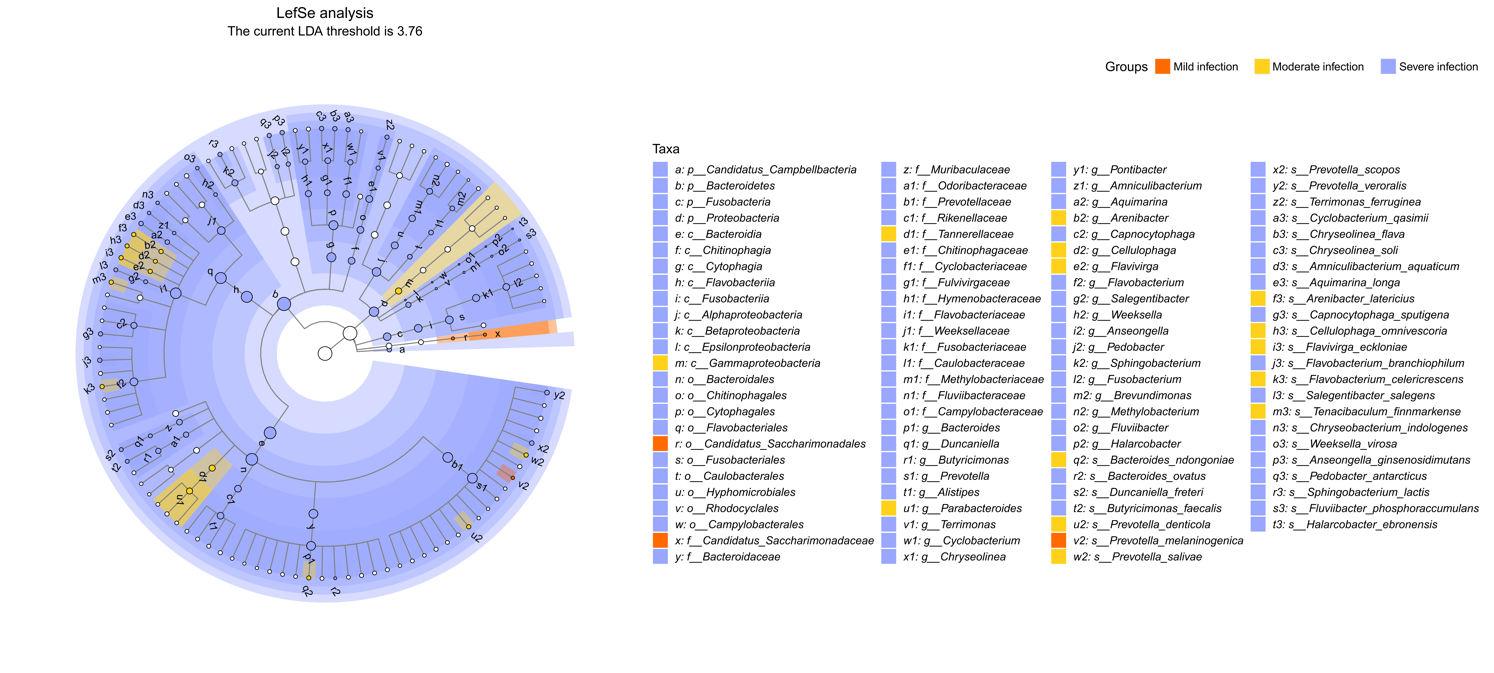
**

**Supplementary Figure 2.** Microbial biomarkers distinguishing different infection severities. This LEfSe cladogram illustrates taxa that are significantly enriched in the Mild (orange), Moderate (yellow), or Severe (blue) infection groups. The hierarchy extends from phylum (inner) to species (outer) levels, and the node size reflects the relative abundance of the taxon.


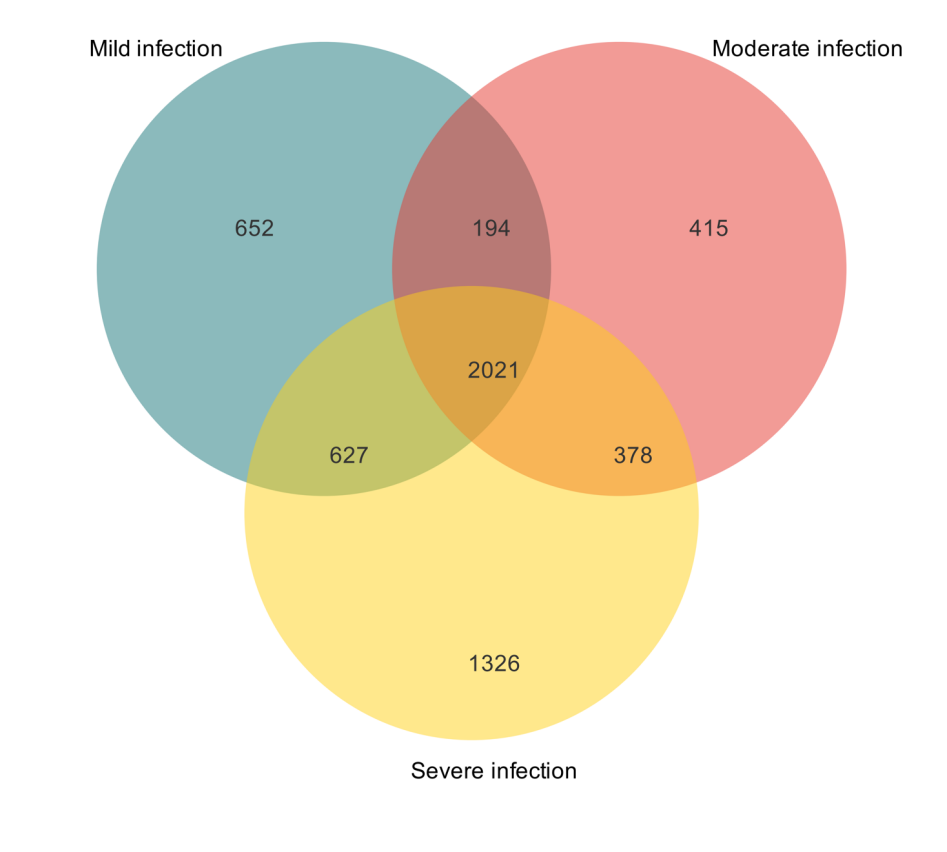


**Supplementary Figure 3.** Venn diagram showing the distribution of bacterial genera among Mild, Moderate, and Severe infection groups. The numbers indicate the count of bacterial genera in each shared or unique section. A core of 2,021 genera was shared across all three groups. The number of genera unique to the Severe infection group (1,326) was notably higher than in the Mild (652) and Moderate (415) groups.


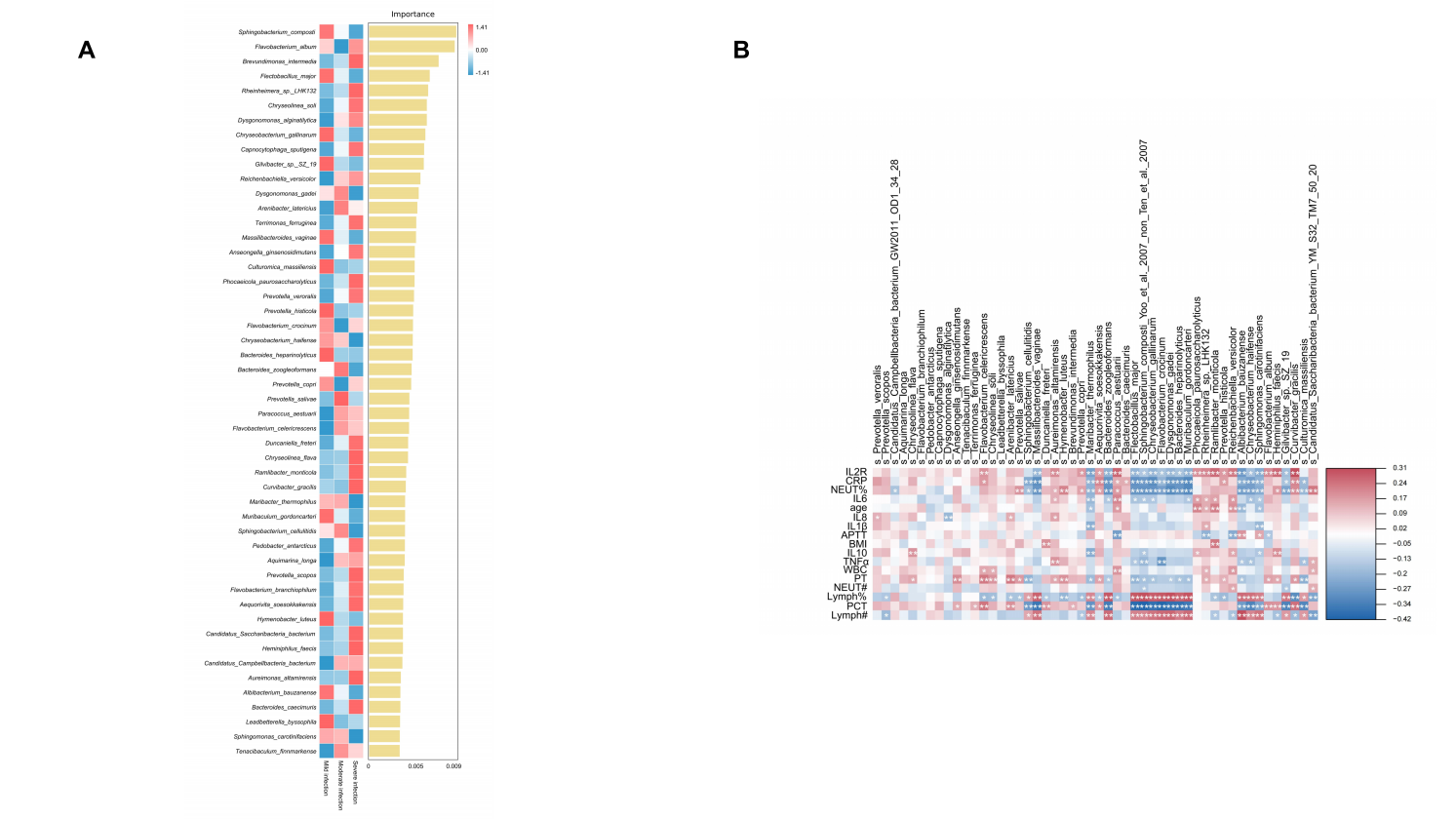


**Supplementary Figure 4.** Species-level analysis identifies key microbial features associated with infection severity**.** (A) Random Forest feature importance analysis showing the importance of different species for distinguishing infection severity. (B) Correlation analysis reveals associations between key microbial genera and host inflammatory status. This heatmap displays the Spearman correlation coefficients between the relative abundances of the most predictive species (identified by Random Forest analysis) and a comprehensive panel of host clinical and laboratory markers. The intensity of the color corresponds to the strength of the correlation (red for positive, blue for negative). Only statistically significant correlations are marked with an asterisk (*q < 0.05; ** q < 0.01).

**Supplementary Table**

**Supplementary Table S1.** Species-Level Characterization of the Genus Streptococcus in OMSI Pus Samples (N=197)

| **Species name** | **Mean Relative Abundance** | **Standard Deviation** | **Detection Count** | **Detection Frequency (N=197)** |
| --- | --- | --- | --- | --- |
| *Streptococcus plurextorum* | 4.75635E-06 | 0.000059215 | 4 | 2.03% |
| *Streptococcus criceti* | 4.56853E-08 | 4.75751E-07 | 2 | 1.02% |
| *Streptococcus peroris* | 4.51777E-07 | 5.39448E-06 | 3 | 1.52% |
| *Streptococcus parauberis* | 1.42132E-07 | 1.41783E-06 | 2 | 1.02% |
| *Streptococcus urinalis* | 1.26904E-07 | 1.25408E-06 | 2 | 1.02% |
| *Streptococcus symci* | 6.54822E-07 | 9.16751E-06 | 1 | 0.51% |
| *Streptococcus thermophilus* | 5.38071E-07 | 7.53299E-06 | 1 | 0.51% |
| *Streptococcus periodonticum* | 2.08122E-07 | 2.25806E-06 | 3 | 1.52% |
| *Streptococcus marmotae* | 1.16751E-07 | 1.19711E-06 | 3 | 1.52% |
| *Streptococcus saliviloxodontae* | 8.62944E-08 | 8.53577E-07 | 2 | 1.02% |
| *Streptococcus porcinus* | 2.99492E-07 | 4.19289E-06 | 1 | 0.51% |
| *Streptococcus ratti* | 1.3198E-07 | 1.46459E-06 | 3 | 1.52% |
| *Streptococcus macedonicus* | 2.03046E-08 | 2.24386E-07 | 2 | 1.02% |
| *Streptococcus mutans* | 1.01523E-08 | 1.42132E-07 | 1 | 0.51% |
| *Streptococcus sp. HSISB1* | 4.60406E-06 | 5.13263E-05 | 4 | 2.03% |
| *Streptococcus thoraltensis* | 3.5533E-08 | 3.54459E-07 | 2 | 1.02% |
| *Streptococcus ruminantium* | 3.80711E-07 | 4.98141E-06 | 3 | 1.52% |
| *Streptococcus halotolerans* | 5.07614E-09 | 7.1066E-08 | 1 | 0.51% |
| *Streptococcus pasteurianus* | 1.77665E-07 | 1.77801E-06 | 3 | 1.52% |
| *Streptococcus sobrinus* | 5.07614E-08 | 5.85321E-07 | 2 | 1.02% |
| *Streptococcus halitosis* | 5.07614E-09 | 7.1066E-08 | 1 | 0.51% |
| *Streptococcus phocae* | 4.56853E-08 | 5.72592E-07 | 2 | 1.02% |
| *Streptococcus pseudoporcinus* | 1.01523E-07 | 1.42132E-06 | 1 | 0.51% |
| *Streptococcus sanguinis* | 3.04569E-08 | 3.17167E-07 | 2 | 1.02% |
| *Streptococcus australis* | 2.03046E-08 | 2.24386E-07 | 2 | 1.02% |
| *Streptococcus dysgalactiae* | 5.07614E-09 | 7.1066E-08 | 1 | 0.51% |
| *Streptococcus loxodontisalivarius* | 3.75635E-07 | 4.98129E-06 | 2 | 1.02% |
| *Streptococcus ursoris* | 1.3198E-07 | 1.30708E-06 | 2 | 1.02% |
| *Streptococcus acidominimus* | 2.03046E-08 | 2.84264E-07 | 1 | 0.51% |
| *Streptococcus anginosus* | 1.52284E-08 | 2.13198E-07 | 1 | 0.51% |
| *Streptococcus chenjunshii* | 5.07614E-09 | 7.1066E-08 | 1 | 0.51% |
| *Streptococcus koreensis* | 3.5533E-08 | 4.97462E-07 | 1 | 0.51% |
| *Streptococcus porci* | 7.46193E-07 | 9.89162E-06 | 2 | 1.02% |
| *Streptococcus pyogenes* | 4.56853E-08 | 5.72592E-07 | 2 | 1.02% |
| *Streptococcus timonensis* | 6.09137E-08 | 8.52792E-07 | 1 | 0.51% |
| *Streptococcus alactolyticus* | 3.65482E-07 | 4.97592E-06 | 2 | 1.02% |
| *Streptococcus constellatus* | 1.52284E-08 | 2.13198E-07 | 1 | 0.51% |
| *Streptococcus caballi* | 1.52284E-08 | 2.13198E-07 | 1 | 0.51% |
| *Streptococcus pneumoniae* | 2.53807E-08 | 2.92661E-07 | 2 | 1.02% |
| *Streptococcus troglodytae* | 4.92386E-07 | 6.6138E-06 | 2 | 1.02% |
| *Streptococcus azizii* | 5.07614E-09 | 7.1066E-08 | 1 | 0.51% |
| *Streptococcus bovimastitidis* | 2.53807E-08 | 2.92661E-07 | 2 | 1.02% |
| *Streptococcus parasanguinis* | 4.06091E-08 | 5.68528E-07 | 1 | 0.51% |
| *Streptococcus pseudopneumoniae* | 2.53807E-08 | 2.92661E-07 | 2 | 1.02% |
| *Streptococcus uberis* | 4.06091E-08 | 5.68528E-07 | 1 | 0.51% |
| *Streptococcus varani* | 4.06091E-08 | 5.68528E-07 | 1 | 0.51% |
| *Streptococcus xiaochunlingii* | 4.06091E-08 | 5.68528E-07 | 1 | 0.51% |
| *Streptococcus sp. A12* | 2.03046E-08 | 2.84264E-07 | 1 | 0.51% |
| *Streptococcus sp. HSISS2* | 4.06091E-08 | 5.68528E-07 | 1 | 0.51% |
| *Streptococcus sp. HSISS3* | 3.5533E-08 | 3.54459E-07 | 2 | 1.02% |
| *Streptococcus agalactiae* | 5.07614E-09 | 7.1066E-08 | 1 | 0.51% |
| *Streptococcus intermedius* | 5.07614E-09 | 7.1066E-08 | 1 | 0.51% |
| *Streptococcus canis* | 5.07614E-09 | 7.1066E-08 | 1 | 0.51% |
| *Streptococcus parasuis* | 2.03046E-08 | 2.84264E-07 | 1 | 0.51% |
| *Streptococcus pharyngis* | 2.03046E-08 | 2.84264E-07 | 1 | 0.51% |
| *Streptococcus pluranimalium* | 2.03046E-08 | 2.84264E-07 | 1 | 0.51% |
| *Streptococcus respiraculi* | 2.03046E-08 | 2.84264E-07 | 1 | 0.51% |
| *Streptococcus rubneri* | 2.03046E-08 | 2.84264E-07 | 1 | 0.51% |
| *Streptococcus salivarius* | 2.03046E-08 | 2.84264E-07 | 1 | 0.51% |
| *Streptococcus sinensis* | 2.03046E-08 | 2.84264E-07 | 1 | 0.51% |
| *Streptococcus suis* | 2.03046E-08 | 2.84264E-07 | 1 | 0.51% |
| *Streptococcus vestibularis* | 2.03046E-08 | 2.84264E-07 | 1 | 0.51% |
| *Streptococcus sp. HSISM1* | 3.54822E-06 | 4.96751E-05 | 1 | 0.51% |
| *Streptococcus sp. NPS 308* | 2.03046E-08 | 2.84264E-07 | 1 | 0.51% |
| *Streptococcus sp. oral taxon 061* | 2.03046E-08 | 2.84264E-07 | 1 | 0.51% |
| *Streptococcus sp. oral taxon 431* | 2.03046E-08 | 2.84264E-07 | 1 | 0.51% |
| *Streptococcus sp. VT 162* | 2.03046E-08 | 2.84264E-07 | 1 | 0.51% |

**Note:** Summary statistics were calculated across the entire cohort (N=197), with zero values included in mean and standard deviation calculations. Detection was defined as relative abundance > 0. The heterogeneity observed across Streptococcus species reinforces the conclusion that the genus-level signal in severity prediction arises from the combined contribution of multiple species rather than a dominant pathogen.

**Supplementary Table S2.** Relative Abundance of Key Streptococcus Species Across OMSI Severity Groups.

| **Species name** | **Mild Group (n=90)** | **Moderate Group (n=41)** | **Severe Group (n=66)** |
| --- | --- | --- | --- |
| *Streptococcus anginosus* | 0 | 0 | 2.79 × 10^-8^ |
| *Streptococcus constellatus* | 0 | 0 | 2.09 × 10^-8^ |
| *Streptococcus intermedius* | ND | ND | ND |
| *Streptococcus mitis* | 0 | 0 | 1.39 × 10^-8^ |
| *Streptococcus oralis* | 0 | 0 | 6.96 × 10^-9^ |

Note: This table details the mean relative abundance of specific Streptococcus species, including classic abscess-forming pathogens (e.g., the *S. anginosus* group) and common commensals, stratified by clinical severity. Values of “0” indicate that the species was not detected in any sample within that severity group. “ND” stands for “Not Detected” in the entire cohort. The extremely low abundances (on the order of 10^-8^ to 10^-9^) observed for all listed species in the severe group suggest that these specific organisms were not the primary drivers of infection severity in this cohort, supporting a model of pathogenesis driven by polymicrobial synergy rather than the dominance of a single, canonical pathogen.

**Supplementary Table S3.** Species-Level Predominance in Individual OMSI Patients

| **Sample ID** | **Predominant Genus**  **(from Table 2)** | **Predominant Species** | **Relative Abundance** | **Severity Group** |
| --- | --- | --- | --- | --- |
| Sample-2 | Prevotella | **No single species >30%** | N/A | Mild |
| Sample-11 | Prevotella | *Prevotella veroralis* | 32.86% | Mild |
| Sample-15 | *Prevotella* | *Prevotella veroralis* | 58.44% | Mild |
| Sample-16 | *Prevotella* | *Prevotella veroralis* | 42.91% | Mild |
| Sample-17 | *Prevotella* | *Prevotella veroralis* | 39.11% | Mild |
| Sample-21 | *Prevotella* | *Prevotella veroralis* | 30.68% | Mild |
| Sample-29 | *Prevotella* | *Prevotella veroralis* | 38.17% | Mild |
| Sample-30 | *Prevotella* | **No single species >30%** | N/A | Mild |
| Sample-32 | *Prevotella* | *Prevotella scopos* | 41.22% | Mild |
| Sample-34 | *Prevotella* | *Prevotella veroralis* | 32.96% | MIld |
| Sample-35 | *Prevotella* | *Prevotella veroralis* | 42.80% | Mild |
| Sample-37 | *Prevotella* | *Prevotella scopos* | 35.99% | Mild |
| Sample-40 | *Prevotella* | *Prevotella veroralis* | 56.46% | Mild |
| Sample-43 | *Prevotella* | **No single species >30%** | N/A | MIld |
| Sample-47 | *Prevotella* | *Prevotella scopos* | 39.99% | Mild |
| Sample-50 | *Prevotella* | *Prevotella veroralis* | 50.69% | Mild |
| Sample-56 | *Prevotella* | *Prevotella scopos* | 67.85% | Mild |
| Sample-59 | *Prevotella* | *Prevotella scopos* | 38.42% | Mild |
| Sample-62 | *Prevotella* | *Prevotella veroralis* | 37.38% | Mild |
| Sample-63 | *Prevotella* | *Prevotella veroralis* | 31.48% | Mild |
| Sample-71 | *Prevotella* | *Prevotella veroralis* | 33.74% | Mild |
| Sample-78 | *Prevotella* | **No single species >30%** | N/A | Mild |
| Sample-84 | *Prevotella* | *Prevotella veroralis* | 50.32% | Mild |
| Sample-91 | *Prevotella* | *Prevotella veroralis* | 39.42% | Moderate |
| Sample-92 | *Prevotella* | *Prevotella scopos* | 40.87% | Moderate |
| Sample-94 | *Prevotella* | *Prevotella veroralis* | 43.77% | Moderate |
| Sample-95 | *Prevotella* | **No single species >30%** | N/A | Moderate |
| Sample-100 | *Prevotella* | *Prevotella veroralis* | 68.00% | Moderate |
| Sample-102 | *Prevotella* | **No single species >30%** | N/A | Moderate |
| Sample-104 | *Prevotella* | *Prevotella veroralis* | 30.01% | Moderate |
| Sample-114 | *Prevotella* | *Prevotella veroralis* | 46.53% | Moderate |
| Sample-115 | *Prevotella* | *Prevotella veroralis* | 31.72% | Moderate |
| Sample-122 | *Prevotella* | *Prevotella scopos* | 59.75% | Moderate |
| Sample-124 | *Prevotella* | *Prevotella veroralis* | 59.23% | Moderate |
| Sample-131 | *Prevotella* | *Prevotella veroralis* | 31.92% | Moderate |
| Sample-133 | *Prevotella* | *Prevotella veroralis* | 51.14% | Severe |
| Sample-134 | *Prevotella* | *Prevotella veroralis* | 41.13% | Severe |
| Sample-136 | *Prevotella* | *Prevotella veroralis* | 79.13% | Severe |
| Sample-139 | *Prevotella* | *Prevotella veroralis* | 43.07% | Severe |
| Sample-140 | *Prevotella* | *Prevotella veroralis* | 36.23% | Severe |
| Sample-141 | *Prevotella* | *Prevotella scopos* | 43.25% | Severe |
| Sample-142 | *Prevotella* | **No single species >30%** | N/A | Severe |
| Sample-143 | *Prevotella* | *Prevotella veroralis* | 44.61% | Severe |
| Sample-153 | *Prevotella* | **No single species >30%** | N/A | Severe |
| Sample-154 | *Prevotella* | **No single species >30%** | N/A | Severe |
| Sample-162 | *Prevotella* | *Prevotella veroralis* | 30.33% | Severe |
| Sample-163 | *Prevotella* | *Prevotella veroralis* | 59.45% | Severe |
| Sample-164 | *Prevotella* | *Prevotella veroralis* | 62.01% | Severe |
| Sample-165 | *Prevotella* | **No single species >30%** | N/A | Severe |
| Sample-166 | *Prevotella* | *Prevotella veroralis* | 33.77% | Severe |
| Sample-171 | *Prevotella* | ***No single species >30%*** | N/A | Severe |
| Sample-173 | *Prevotella* | *Prevotella veroralis* | 30.75% | Severe |
| Sample-180 | *Prevotella* | *Prevotella veroralis* | 65.56% | Severe |
| Sample-185 | *Prevotella* | *Prevotella veroralis* | 39.26% | Severe |
| Sample-191 | *Prevotella* | *Prevotella veroralis* | 72.34% | Severe |
| Sample-192 | *Prevotella* | **No single species >30%** | N/A | Severe |
| Sample-195 | *Prevotella* | *Prevotella veroralis* | 83.34% | Severe |
| Sample-196 | *Prevotella* | **No single species >30%** | N/A | Severe |
| Sample-197 | *Prevotella* | *Prevotella veroralis* | 34.75% | Severe |
| Sample-60 | *Chryseolinea* | *Chryseolinea flava* | 33.69% | Mild |
| Sample-113 | *Chryseolinea* | *Chryseolinea flava* | 46.64% | Moderate |
| Sample-132 | *Chryseolinea* | **No single species >30%** | N/A | Severe |
| Sample-147 | *Chryseolinea* | *Chryseolinea flava* | 32.69% | Severe |
| Sample-181 | *Chryseolinea* | *Chryseolinea flava* | 36.98% | Severe |
| Sample-182 | *Chryseolinea* | *Chryseolinea flava* | 54.43% | Severe |
| Sample-183 | *Chryseolinea* | *Chryseolinea flava* | 36.15% | Severe |
| Sample-192 | *Chryseolinea* | *Chryseolinea flava* | 33.33% | Severe |
| Sample-128 | *Flavobacterium* | **No single species >30%** | N/A | Moderate |
| Sample-4 | *Aquimarina* | *Aquimarina longa* | 31.68% | Mild |
| Sample-9 | *Aquimarina* | *Aquimarina longa* | 34.23% | Mild |
| Sample-20 | *Bacteroides* | *Bacteroides stercoris* | 91.71% | Mild |
| Sample-39 | *Bacteroides* | *Bacteroides stercoris* | 82.19% | Mild |
| Sample-45 | *Bacteroides* | *Bacteroides stercoris* | 61.42% | Mild |
| Sample-55 | *Bacteroides* | *Bacteroides stercoris* | 33.62% | Mild |
| Sample-151 | *Bacteroides* | *Bacteroides ovatus* | 40.78% | Severe |
| Sample-196 | *Bacteroides* | *Bacteroides stercoris* | 38.14% | Severe |
| Sample-3 | *Croceivirga* | *Croceivirga lutea* | 32.83% | MIld |
| Sample-8 | *Croceivirga* | *Croceivirga lutea* | 31.45% | Mild |
| Sample-49 | *Croceivirga* | *Croceivirga lutea* | 39.85% | Mild |
| Sample-6 | *Paracoccus* | *Paracoccus lichenicola* | 89.51% | Mild |
| Sample-10 | *Paracoccus* | *Paracoccus lichenicola* | 82.09% | Mild |
| Sample-14 | *Paracoccus* | *Paracoccus lichenicola* | 96.12% | Mild |
| Sample-19 | *Paracoccus* | *Paracoccus lichenicola* | 97.12% | Mild |
| Sample-31 | *Paracoccus* | *Paracoccus lichenicola* | 72.17% | Mild |
| Sample-116 | *Paracoccus* | *Paracoccus lichenicola* | 92.02% | Moderate |
| Sample-189 | *Capnocytophaga* | *Capnocytophaga sputigena* | 41.92% | Severe |
| Sample-23 | *Pedobacter* | *Pedobacter antarcticus* | 36.87% | Mild |
| Sample-38 | *Pedobacter* | *Pedobacter helvus* | 44.48% | Mild |
| Sample-183 | *Pedobacter* | *Pedobacter antarcticus* | 30.71% | Severe |
| Sample-81 | *Dysgonomonas* | *Dysgonomonas alginatilytica* | 30.67% | Mild |
| Sample-82 | *Dysgonomonas* | *Dysgonomonas alginatilytica* | 52.30% | Mild |
| Sample-106 | *Dysgonomonas* | *Dysgonomonas alginatilytica* | 50.30% | Moderate |

Note: This table provides species-level resolution for the 92 samples showing genus-level predominance in Table 2. Each row represents an individual patient sample, detailing the specific bacterial species that exceeded the 30% relative abundance threshold. Samples where no single species reached this threshold within a predominant genus are indicated accordingly.

**
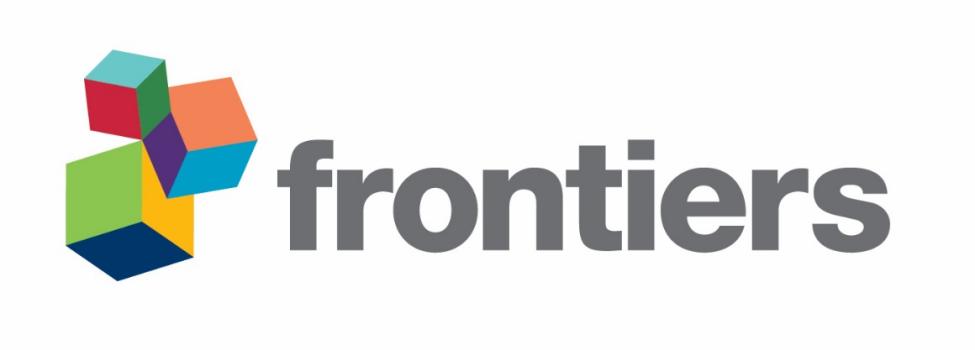
**
